# Prevention of radiation-induced bystander effects by agents that inactivate cell-free chromatin released from irradiated dying cells

**DOI:** 10.1038/s41419-018-1181-x

**Published:** 2018-11-15

**Authors:** Saurabh Kirolikar, Preeti Prasannan, Gorantla V. Raghuram, Namrata Pancholi, Tannishtha Saha, Pritishkumar Tidke, Pradip Chaudhari, Alfina Shaikh, Bhagyeshri Rane, Richa Pandey, Harshada Wani, Naveen K. Khare, Sophiya Siddiqui, Jenevieve D’souza, Ratnam Prasad, Sushma Shinde, Sailee Parab, Naveen K. Nair, Kavita Pal, Indraneel Mittra

**Affiliations:** 1 Translational Research Laboratory, Advanced Centre for Treatment, Research and Education in Cancer, Tata Memorial Centre, Navi-Mumbai, 410210 India; 2 Comparative Oncology Program and Small Animal Imaging Facility, Advanced Centre for Treatment, Research and Education in Cancer, Tata Memorial Centre, Navi-Mumbai, 410210 India

## Abstract

Radiation-induced bystander effect (RIBE) is a poorly understood phenomenon wherein non-targeted cells exhibit effects of radiation. We have reported that cell-free chromatin (cfCh) particles that are released from dying cells can integrate into genomes of surrounding healthy cells to induce DNA damage and inflammation. This raised the possibility that RIBE might be induced by cfCh released from irradiated dying cells. When conditioned media from BrdU-labeled irradiated cells were passed through filters of pore size 0.22 µm and incubated with unexposed cells, BrdU-labeled cfCh particles could be seen to readily enter their nuclei to activate H2AX, active Caspase-3, NFκB, and IL-6. A direct relationship was observed with respect to activation of RIBE biomarkers and radiation dose in the range of 0.1–50 Gy. We confirmed by FISH and cytogenetic analysis that cfCh had stably integrated into chromosomes of bystander cells and had led to extensive chromosomal instability. The above RIBE effects could be abrogated when conditioned media were pre-treated with agents that inactivate cfCh, namely, anti-histone antibody complexed nanoparticles (CNPs), DNase I and a novel DNA degrading agent Resveratrol-copper (R-Cu). Lower hemi-body irradiation with γ-rays (0.1–50 Gy) led to activation of H2AX, active Caspase-3, NFκB, and IL-6 in brain cells in a dose-dependent manner. Activation of these RIBE biomarkers could be abrogated by concurrent treatment with CNPs, DNase I and R-Cu indicating that activation of RIBE was not due to radiation scatter to the brain. RIBE activation was seen even when mini-beam radiation was delivered to the umbilical region of mice wherein radiation scatter to brain was negligible and could be abrogated by cfCh inactivating agents. These results indicate that cfCh released from radiation-induced dying cells are activators of RIBE and that it can be prevented by treatment with appropriate cfCh inactivating agents.

## Introduction

Radiation-induced bystander effect (RIBE) is a phenomenon wherein cells not directly exposed to ionizing radiation show heritable changes that include DNA damage, mutations, chromosomal aberrations, chromosomal instability, senescence, apoptosis, and oncogenic transformations^1,2^. Although RIBE has been well documented in a variety of biological systems, the mechanism(s) by which RIBE is activated is not well understood. It is thought that multiple pathways are involved in the bystander phenomenon, and different cell types respond differently to bystander signaling^1,2^.

Inter-cellular gap-junctional communication or soluble factors released from irradiated cells have been implicated in RIBE^3,4^. Experiments in vitro have shown that filtered conditioned media from irradiated cells induce RIBE when added to un-irradiated cells^5^. Reactive oxygen species (ROS)^6^ and secondary messengers, such as nitric oxide (NO)^7^, protein kinase^8^ as well as cytokines, such as TGF-β^9^ and TNF-α^10^ have also been considered to be involved in RIBE. Bystander effects have been reported using synchrotron—generated micro—beam irradiation^11,12^, and targeted cytoplasmic irradiation has been shown to induce bystander responses^13^, challenging the belief that direct damage to DNA is a prerequisite for RIBE. In addition to DNA damage and apoptosis, high dose micro-beam irradiation has been reported to generate local and systemic immune responses^12^. Recent reports suggest that miRNAs play an important role in inter-cellular signaling between irradiated and bystander cells^14,15^. Serum from patients who have received focal radiation therapy have been shown to have RIBE-inducing properties, and out-of-field RIBE has been reported in distant organs^16^. Evidence of RIBE was demonstrated in non-small cell lung cancer patients exposed to focal irradiation wherein DNA damage was observed in both irradiated and out-of-field normal cells^17^. Cranial X-irradiation of mice has been reported to lead to elevated DNA damage, altered cellular proliferation, apoptosis, and increased p53 levels in the shielded spleen^18^. Development of brain tumors in susceptible strains of mice exposed to trunk irradiation is another example of RIBE induced in distant organs^19^. Evidence of RIBE in the form of clastogenic effects and elevated levels of micronuclei, signifying DNA damage, was observed when cells were exposed to sera from victims of Chernobyl disaster long after exposure to ionizing radiation^20^. However, in spite of extensive research demonstrating the phenomenon of RIBE in various biological systems and identification of multiple agents involved in inter-cellular signaling, the mechanism(s) responsible for RIBE are still not fully understood^1,2^.

Apoptotic cell death with release of nucleosomes is one of the hallmarks of cell death following ionizing radiation^21,22^. We have recently reported that cfCh particles (nucleosomes) that are released from dying cells can integrate into surrounding healthy cells to induce DNA damage and inflammation^23^. We have also reported that cfCh derived from dying cells that circulate in blood can have systemic damaging effects on cells of the host^24,25^. They can incorporate themselves into host cell genomes and induce dsDNA breaks and apoptosis of healthy cells^23–25^. These findings led us to hypothesize that RIBE may be activated by cfCh that are released from dying cells exposed to ionizing radiation by integrating themselves into genomes of neighboring un-irradiated cells, or those of distant organs, to damage their DNA.

## Materials and methods

### Cell lines

NIH3T3 (mouse fibroblast), Jurkat (human lymphoblastic leukemia), and MDA-MB-231 (human breast cancer) cells were obtained from the American Type Culture Collection (ATCC), USA. NIH3T3 and Jurkat cells were grown in Dulbecco’s Modified Eagle’s Medium (DMEM) (Gibco, catalog#12800-017) containing 10% bovine calf serum (HyClone, catalog#SH30073), whereas MDA-MB-231 cells were cultured in DMEM containing 10% fetal bovine serum (Gibco, catalog#26140079). Cells were maintained in an incubator at 37 °C in an atmosphere of 5% CO_2_ and air.

### Experiments to investigate bystander uptake of fluorescently dual-labeled cfCh

Jurkat cells were plated at a density of 6 × 10^4^ cells and after overnight culture (cell count 1 × 10^5^) were dually labeled in their histones (H2B) and DNA. Histone H2B labelling was done for 36 h with CellLight® Histone 2B-GFP (Thermo Fisher Scientific, MA, USA, Catalogue No. C10594) and DNA labeling for 24 h using BrdU (10 μM Sigma Chemicals, MO, USA, Catalogue No. B5002). Dually labeled Jurkat cells were plated at a density of 6 × 10^4^, and after overnight culture (~16 h) when the cell density was ~1 × 10^5^, they were irradiated using a telecobalt machine (Bhabatron-II)^©^ delivering ~2.26 Gy/min to a total dose of 15 Gy. Irradiated Jurkat cells were co-cultivated with NIH3T3 cells in a ratio of 1:1 in 35 mm^3^ Petri dishes for 24 h. Cells were thoroughly washed and the adherent bystander NIH3T3 cells were processed for confocal microscopy.

### Experiments to investigate RIBE using BrdU-labeled irradiated cells

We employed the experimental system using filtered conditioned medium to investigate whether cfCh were involved in RIBE^3,26^ NIH3T3 cells pre-labeled for 24 h with 10 μM BrdU were seeded in 35 mm plates (6 × 10^4^), and after overnight culture (~16 h) when the cell density was ~1 × 10^5^, they were irradiated using a tele Cobalt machine (Bhabatron-II)^©^ delivering ~2.26 Gy/min to a total dose of 10 Gy. Cells were returned to the incubator and after 6 h, conditioned media from donor cells were collected and passed through syringe filters comprising of pore sizes of 0.22 µm and 0.1 µm (Pall Corporation, Catalog # PN4612 and PN4611, respectively). The filtered conditioned media were applied to un-irradiated NIH3T3 cells plated on cover slips in 35 mm culture dishes (1 × 10^5^ cells). After 12 h of incubation, the recipient cells were washed, fixed, and processed for immunofluorescence to detect presence of fluorescent BrdU particles in the recipients and activation of RIBE in the form of expression of γ-H2AX, active Caspase-3, NFκB, and IL-6 (see later for immunofluorescence methodology).

### Experiments to investigate RIBE in different cellular combinations

The same experimental protocol described above was used except that the irradiated cells were not labeled with BrdU and the filtered culture medium was applied to recipient bystander cells for 6 h. The various cellular combinations used comprising (1) NIH3T3 (mouse normal donor) and NIH3T3 (mouse normal recipient); (2) MDA-MB-231 (human tumor donor) and NIH3T3 (mouse normal recipient); (3) MDA-MB-231 (human tumor donor) and MDA-MB-231 (human tumor recipient); (4) NIH3T3 (mouse normal donor) and MDA MB-231 (human tumor recipient). Various RIBE parameters namely γH2AX, active caspase-3, NFκB, and IL-6 were analyzed by immuno-flourescence.

### Electron microscopy to detect cfCh in conditioned media

The filtered conditioned medium (~1.5 ml) from cells of six irradiated MDA-MB-231 culture dishes were pooled and centrifuged at 700,000×*g* for 16 h, and the pellet obtained was washed in PBS by another centrifugation at 700,000×*g* for 16 h. Un-filtered cultured media were similarly processed and used as controls. The resulting pellets were re-suspended in 50 µl PBS and 10 µl aliquots were processed for negative staining technique as described by Horne, 1965^27^. Grids layered with the samples were examined under JEM1400 transmission electron microscope and imaged at × 60,000 magnification^28^.

### Dosimetry for in vitro studies

Cells were plated in six-well plates and exposed to a radiation dose of 10 Gy (Supplementary Fig. 1). The field area was 25 × 25 cm^2^ covering the entire plate (12.5 × 8.5 cm). The depth of irradiation was kept at 0.5 cm and the exposure was given for 5.89 min to deliver 10 Gy. The plate was placed exactly at the center of the field (away from the prenumbra) so as to obtain uniform radiation exposure.

### Estimation of cell death

Estimation of cell death was performed using the acridine orange (AO)/propidium iodide (PI) staining method by fluorescence microscopy. AO is a membrane-permeable dye that stains DNA of healthy cells while PI stains DNA of dead or dying cells. Following radiation at different doses (0.1 Gy, 0.25 Gy, 0.5 Gy, 0.75 Gy, 1 Gy, 2.5 Gy, 5 Gy, 10 Gy, and 50 Gy), MDA-MB-231 cells were incubated for 6 h and were stained at 1:1 ratio of AO and PI and mounted with VectaShield mounting medium with DAPI (Vector Laboratories, Catalog #H-1200). Images were acquired immediately and analyzed on Applied Spectral Imaging system. A total of 1000 nuclei staining with either dye were counted and proportion of nuclei that stained with PI were scored as dead. PI was sourced from Sigma-Aldrich Merck, Catalog # P4170 while AO was a kind gift from Dr. S Chiplunkar, ACTREC.

### Apoptosis or necrosis

To determine what proportion of cell death was due to apoptosis and/or necrosis, irradiated cells (10 Gy) were incubated for 6 h post irradiation and were concurrently stained with PI and active caspase-3 (a marker of apoptosis) or PI and cyclophilin-A (a marker of necrosis). Proportion of nuclei that stained with active caspase-3 or cyclophilin-A that co-localized with PI stained nuclei were counted (Supplementary Fig. 4).

### Chemotherapy-induced bystander effect

MDA-MB-231 cells were treated with Adriamycin (5 µg/ml for 24 h). Cells were trypsinised and extensively washed in PBS and incubated in fresh medium for 12 h. The conditioned medium was passed through 0.22 µm filter and applied to NIH3T3 cells for 6 h. Activation of H2AX was estimated by immuno-flourescence.

### Isolation of cfCh from irradiated conditioned medium

cfCh from filtered conditioned media of irradiated (10 Gy) MDA-MB-231 cells that had been pre-labeled in their DNA with BrdU and histones with CellLight® Histone 2B-GFP were isolated by a method described us in detail elsewhere^24^. The purification method primarily comprises of ultra-centrifugation, lysis and affinity chromatography^24^. The final isolate was suspended 500 µl of PBS, and 100 µl from the suspension was cytospun on a slide and examined by confocal microscopy. In a parallel set of experiments, cfCh were similarly isolated from irradiated filtered conditioned media that had not been fluorescently dually labeled and were similarly applied on a slide and stained with exosome component marker (Hsp 70) to confirm that cfCh isolates were devoid of exosomes.

### Experiments using cfCh inactivating agents

In order to further confirm that cfCh are involved in RIBE, we treated the irradiated filtered conditioned medium with cfCh neutralizing/degrading agents (described below) prior to applying them to un-irradiated bystander cells. We used four different combinations of cells as mentioned earlier. The cfCh inactivating agents used were: (1) anti-histone antibody complexed nanoparticles (CNPs) (25 µg/1.5 ml)^29^, DNase I (0.0005 U/ml, Sigma, Catalog#DN25) or a novel DNA degrading agent resveratrol-copper (R-Cu) (molar ratio of 1 mM R: 0.0001 mM Cu) (see later)^30^.

### Immunofluorescence

Primary and secondary antibodies used in the immunofluorescence experiments are listed in Supplementary Table 1.

#### In vitro experiments

Immunofluorescence to detect presence of BrdU and activation of H2AX, active Caspase-3, NFκB, and IL-6 was performed by standard procedure described by us earlier^23,24^. Slides were mounted with vectashield mounting medium with DAPI (Vector Laboratories, Catalog#H-1200) and analyzed on Applied Spectral Imaging system. All experiments were done in duplicate; 200 cells were analyzed for each cellular combination and mean fluorescence intensity (MFI) was calculated and comparison between groups was performed using Student’s *t*-test. In case of γ-H2AX and NFκB, nuclear fluorescence was used to calculate MFI.

#### In vivo experiments

The in vivo experiments consisted of delivering lower hemi-body irradiation (HBI) or focused mini - beam irradiation details of which are described later. Cryo-sections of brains of control and irradiated animals were immuno-stained for detection of γH2AX, active Caspase-3, NFκB, and IL-6 using primary and secondary antibodies described above. One thousand cells were analyzed from each brain and results were expressed as mean MFI (±S.E.). Comparison between groups was performed using Student’s *t*-test. Activation of the above RIBE markers appeared as fluorescent foci. We ascertained at the outset that fluorescent foci count strictly correlated with MFI values and expressed the results as such. To confirm that DNA damage (γH2AX) was confined to neuronal cells (astrocytes), antibody against Glial Fibrillary Acidic Protein (GFAP) was used, which were applied on 3 µm paraffin sections. The secondary antibody was donkey anti-rabbit TRITC (Supplementary Fig. 12).

### Fluorescence in situ hybridization (FISH)

Metaphase spreads were prepared from bystander NIH3T3 cells (at 10th passage) treated with conditioned medium of irradiated and un-irradiated MDA-MB-231 cells. Metaphases were hybridized with Cyanine-3 orange labeled human whole genomic probe (Chrom-Bios GmbH, Germany. Catalogue No. HGDNAOR10). Thirty metaphases were imaged in each case and examined for the presence of human DNA signals using FISHview software 5.0 (Applied Spectral Imaging, Israel).

### Analysis of chromosomal aberrations

Metaphase spreads were prepared at 10th passage from recipient NIH3T3 cells that had been treated with conditioned medium from irradiated or un-irradiated MDA-MB-231 cells and stained through conventional Giemsa staining. Fifty metaphases were analyzed in each case and distribution and frequency of chromosomal aberrations were analyzed using the Bandview software 5.0 on Spectral Bio-Imaging System (Applied spectral imaging, Israel).

### Hemi-body irradiation to investigate RIBE in vivo

BALB/c mice, aged 6–8 weeks, were subjected to lower HBI at different doses viz, 0.1 Gy, 0.5 Gy, 1 Gy, 2.5 Gy, 5 Gy, and 10 Gy wherein the upper half of the body was covered with lead shield. Each radiation group comprised of five animals. Lower HBI was delivered by gamma radiation using a Cobalt-60 source by single anterior portal of 20 cm × 5 cm field size. The radiation doses were normalized at 1.5 cm depth and at a dose rate of 175 cGy/minute. The HBI setup is depicted in Supplementary Fig. 2. The animals were killed after 48 h and their brains were removed, snap-frozen, and subsequently processed for cryo-sectioning and immune-florescence staining. Studies using cfCh inhibitors were undertaken in animals receiving 10 Gy radiation dose. Twenty-five animals were divided into 5 groups of five animals each as follows: (1) control, (mice not exposed to radiation); (2) 10 Gy lower HBI alone; (3) 10 Gy lower HBI+CNPs (50 µg anti-H4 conjugated nanoparticles once daily i.p.); (4) 10 Gy lower HBI+DNase I (15 mg/kg twice daily i.p.); (5) 10 Gy lower HBI+R-Cu (*R* = 1 mg/kg and Cu = 10^−4^mg/kg twice daily by oral gavage). The first dose of CNPs, DNase I and R–Cu were administered 4 h prior to delivery of lower HBI; subsequent doses and frequency of administration of cfCh inhibitors are given in appropriate sections below.

### Dosimetry of hemi-body irradiation experiments

Thermo-luminescent dosimeters (TLDs) were prepared in-house using 40 mg of freshly annealed TLD-100 (LiF: Mg,Ti) powder (Harshaw Chemical Co. Solon, Ohio USA) and packed in square polyethylene pouches (1 cm × 1 cm). BALB/c mice were killed under CO_2_ atmosphere and TLD pouches were surgically placed close to various organs/tissue namely thigh muscle, liver, lung, heart, and brain. Nylon sutures were used for closure of incisions. Mice (*n* = 2 per dose) were exposed to various radiation doses as described earlier. TLDs were collected post irradiation for dosimetry analysis. The thermoluminescent output was recorded using commercial TLD-reader (REXON UL-320) and expressed as output per unit weight (nC/mg). The uncertainty factor in TLD-100 powder measurements is ±2%. Dosimtery data are given in Supplementary Table 2.

### Mini beam irradiation using Varian/BrainLAB Novalis Tx™ radiotherapy platform

Clinical Varian Novalis Tx™ 6 MV accelerator powered by TrueBeam STx was used for focused beam irradiation. This platform has capabilities for targeted Stereotactic Body Radiation Therapy and includes Brainlab iPlan® treatment planning and Varian’s HD120 MLC multileaf collimator for high resolution beam shaping. BalbC mice, aged 6–8 weeks, were subjected for focused mini beam radiation of 1 cm diameter at 2000 cGy dose over the umbilical region under general anesthesia (Supplementary Fig. 3). Target to surface distance (TSD) technique was used with TSD at surface 100 cm for dose delivery. Animals were placed in supine position and dose was delivered with 1.5 cm depth of prescription. Dose exposure plan was generated using Varian Eclipse planning system (v 13). Varians HD MLC were used to shape the treatment diameter of 10 mm and planned to deliver a 20 Gy of dose to the target. A wax bolus of 8 mm thickness was used to create sufficient dose build-up. The scatter radiation dose for target to non-target regions is depicted in Supplementary Fig. 3.

### Dosimetry for focused mini-beam irradiation experiments

The calibrated chambers and radiochromic film were used for scattered dose calculations in non-target region. Solid water slab phantom was used to check the accuracy of the Plan generated by the treatment planning system. Plan-specific quality assurance were performed before the actual delivery of dose. Focused mini-beam radiation of 1 cm diameter at 2000 cGy dose over the umbilical region produce a scatter to the brain which was equivalent to that in ambient atmosphere (0.01216 Gy) (Supplementary Fig. 3). Forty-eight hr after radiation, mice were killed under anesthesia and brain tissue were collected and cryopreserved. The cryosections of brain tissue were analyzed for activation of H2AX and NFkB expression by Immuno-flurescence.

### Agents used to inactivate cfCh

#### Anti-histone antibody complexed nanopaticles (CNPs)

Synthesis of pullulan-histone antibody nanoconjugates was performed as described by us earlier using H4 IgG^23,24,29^. For in vitro studies, 25 µg of CNPs in 100 µl of buffer was added to 1.5 ml of culture medium. For in vivo studies, 50 µg of CNPs in 100 µl of buffer was administered i.p. once a day for a duration of 48 h when the animals were killed (total number of doses received = 3). The first dose of CNPs was given 4 hr prior to irradiation.

#### Resveratrol-copper (R-Cu)

Resveratrol (R) is a plant polyphenol with antioxidant properties^31^. However, R acts as a pro-oxidant in the presence of copper (Cu) by reducing Cu (II) to Cu (I) thereby generating a free radical^32^. R-Cu can cleave plasmid DNA by this pro-oxidant activity^33^. We have shown that R-Cu can also degrade genomic DNA^30^, and that the pro-oxidant property of R-Cu with respect to degradation of DNA is maintained even when the molar concentration of Cu is substantially reduced with respect to that of R^30^.

#### In vitro experiments

The R-Cu molar combination used in these experiments was R (1 mM): Cu (0.0001 mM). R (2.3 mg) (Sigma, Catalog#R5010) was dissolved in 5 ml of 30% ethanol (2 mM) (solution A). Copper sulphate (4.98 mg) (MP Biomedicals, Catalog#191415) was dissolved in 1 ml distilled water (20 mM) and then serially diluted to 0.0002 µM concentration (solution B). Solutions A and B were mixed (50% v/v) to obtain a mixture containing 1 mM R and 0.0001 mM Cu. One hundred microlitres of this mixture was added to 1.5 ml of culture medium.

#### In vivo experiments

We used resveratrol (Trade name—TransMaxTR, Biotivia LLC, USA) and copper (Trade name—Chelated Copper, J.R. Carlson Laboratories Inc. USA) for in vivo experiments, both of which are approved for human consumption as dietary supplements. The contents of 500 mg capsules of R were dissolved in sterile distilled water (concentration = 0.4 mg/ml). Five milligram tablets of copper were crushed into fine powder and dissolved in distilled water (concentration = 0.04 µg/ml). Both solutions were administered (50 µl each) by oral gavage one followed by the other twice daily for a duration of 48 h when they were killed (total number of doses received = 5). The final concentration of R was 1 mg/kg and Cu was 0.1 µg/kg at a final ratio of 1:10−4. The first dose of R-Cu was given 4 hr prior to irradiation.

#### DNase I

##### In vitro experiments

Bovine pancreatic DNase I (0.005 U; Sigma-Aldrich; Catalogue No- DN25-1G) was used per 1.5 ml of culture media in all experiments.

##### In vivo experiments

Bovine pancreatic DNase I (Sigma-Aldrich; Catalogue No- DN25-1G) was injected i.p. at 15 mg/kg twice daily for a duration of 48 h when they were killed (total number of doses received = 5). The first dose of DNase I was given 4 hr prior to irradiation.

### Estimation of serum cfCh

Mice were irradiated with lower HBI (10 Gy) and serial blood samples were collected at 12 h, 24 h, 36 h, 48 h, and 60 h. Serum was separated and serum cfCh was estimated by Cell Death Detection ELISAplus kit (Roche Diagnostics GmbH, Germany) as described by us earlier^24^. Results are expressed in terms of absorbance kinetics at 405 nm.

### Animal ethics approval

The protocol for animal experiments were approved by the Institutional Animal Ethics Committee (IAEC) of the Institute in compliance to ARRIVE guidelines. C57Bl6 and BALB/c mice (6–8 weeks old weighing ~20 g) were obtained from and housed in the Institute Animal House Facility.

### Statistical analysis

GraphPad Prism 5 (GraphPad Software, Inc., USA. Version 5.0) was used to perform statistical analysis. Results were compared using Student’s *t*-test. Graphs were presented as mean ± standard error of mean (SEM). Differences between groups were considered significant when *P*-value was <0.05 (two-tailed).

## Results

**Fig. 1:**
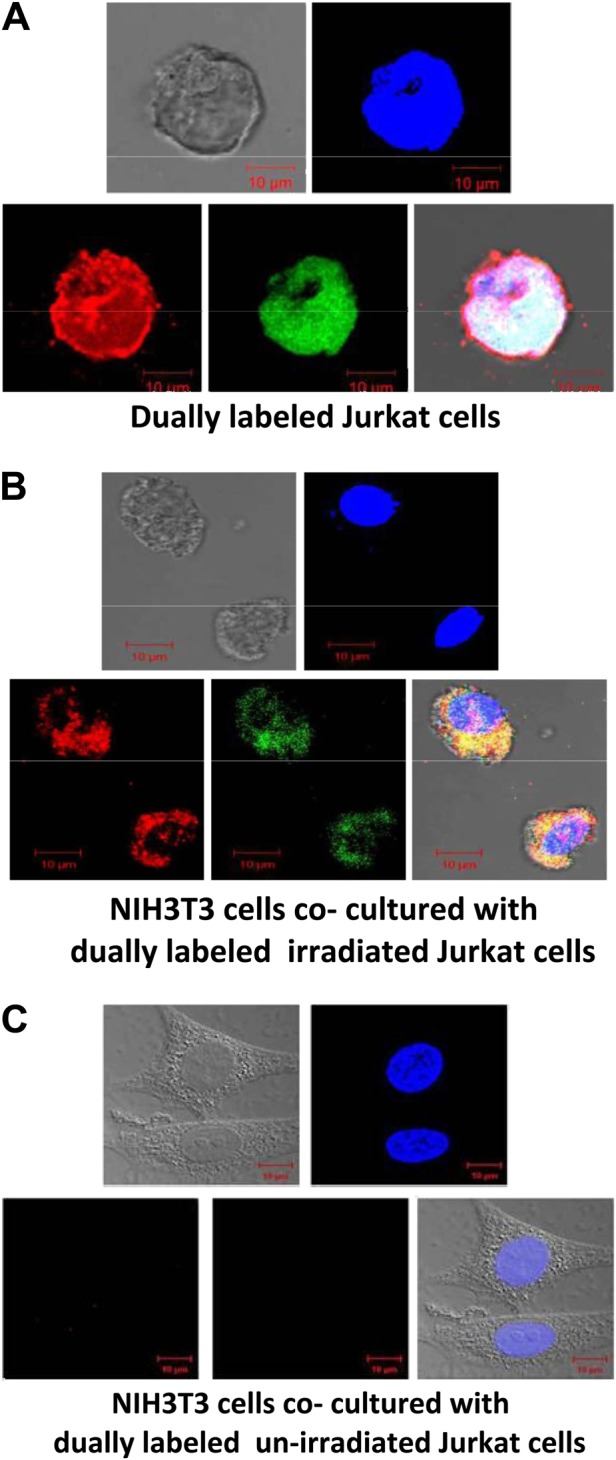
Confocal microscopy images to demonstrate bystander uptake by NIH3T3 cells of dually labeled cfCh released from irradiated dying Jurkat cells that had been labeled in their DNA with BrdU (red) and histones by CellLight® Histone 2B-GFP (green). **A** Dually labeled Jurkat cells. **B** NIH3T3 cells that had been co-cultivated with irradiated (15 Gy) dually labeled Jurkat cells for 24 h. **C** NIH3T3 cells that had been co-cultivated with un-irradiated dually labeled Jurkat cells for 24 h

Radiation is known to induce apoptotic cell death with release of nucleosomes/chromatin fragments in the culture medium^21,22^. A confocal image of Jurkat cells that had been dually pre-labeled in their DNA (BrdU) and H2B histone (Celllight® Histone 2B-GFP) is given in Fig. 1A. When the dually labeled Jurkat cells were administered 15 Gy of radiation and co-cultured with NIH3T3 cells for 24 h, numerous dually labeled cfCh particles were observed in the cytoplasm and nuclei of the NIH3T3 cells (Fig. 1B). The fluorescent intensity of the dual labeled Jurkat cell shown in Fig. 1A was 107.13, whereas the mean fluorescence intensity (MFI) of the two NIH3T3 cells (Fig. 1B) was 27.86 ± 1.79 (mean ± SE). This suggested that 26% of the dually labeled fluorescent cfCh particles had been transferred to the bystander NIH3T3 cells. Little uptake of labeled particles were observed when un-irradiated Jurkat cells were co-cultivated with NIH3T3 cells (Fig. 1C).

**Fig. 2:**
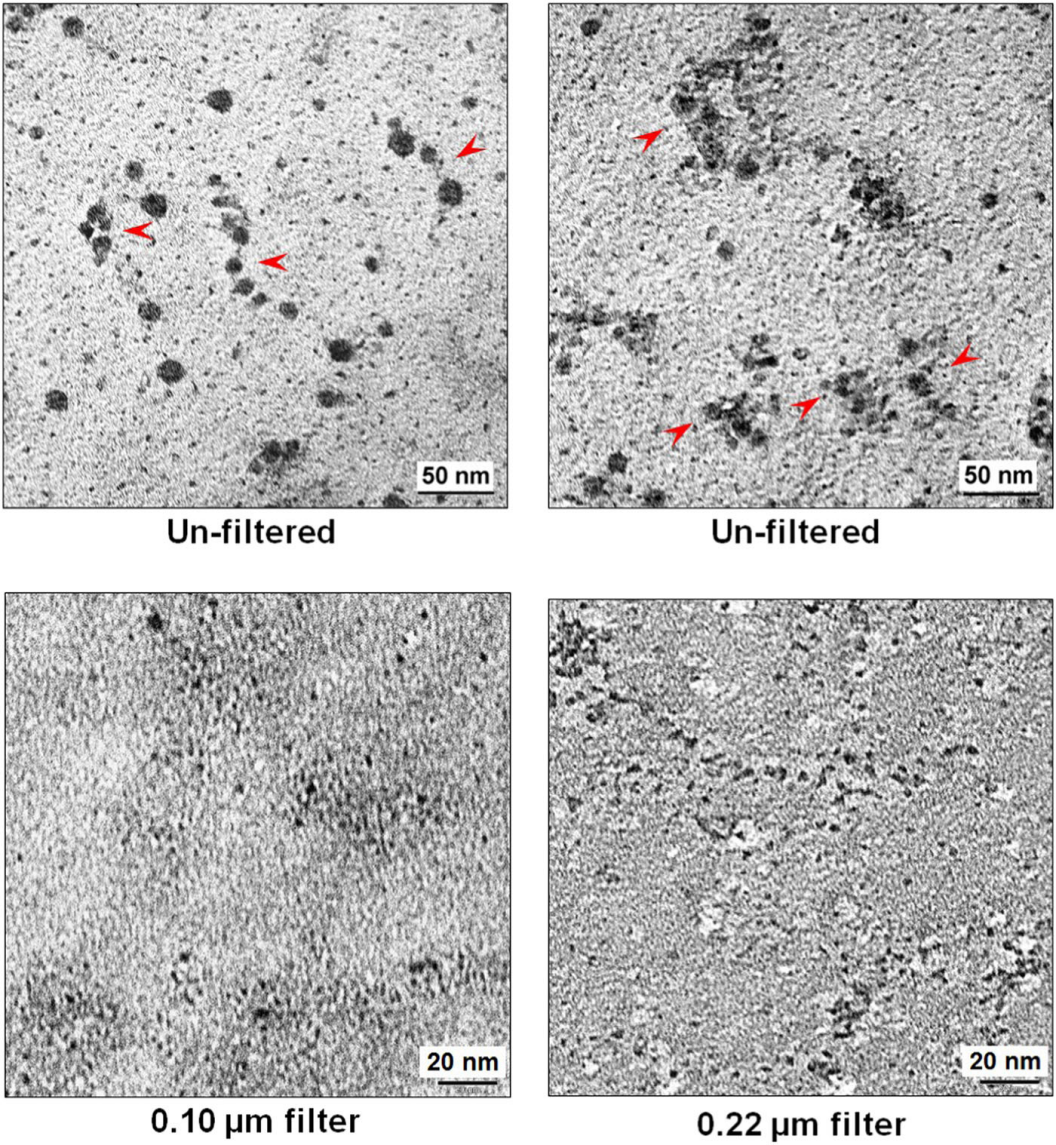
EM images of un-filtered and filtered irradiated conditioned media of MDA-MB-231 human breast cancer cells. Images in the upper panel are of un-filtered media, which show ‘beads on a string’ structures typical of chromatin. These structures are either discrete (left hand image) or are seen in clumps (right hand image). The observed nucleosomes are ~10 nm in size. Filtered conditioned media, however, suggest that the mechanical force applied during filtration through small pores 0.1 µm and 0.22 µm in size has broken up the chromatin-like structures and the particles seen were sub-nucleosomal in size (<10 nm) (lower panels)

We next undertook EM studies of both filtered and un-filtered conditioned medium of irradiated MDA-MB-231 cells to investigate whether cfCh particles could be visually demonstrated. EM of un-filtered conditioned media showed ‘beads on a string’ like structures typical of chromatin (Fig. 2, upper panels). These structures were either discrete (upper left hand image) or were seen in clumps (upper right hand image). Images of filtered conditioned media, however, suggested that the mechanical force applied during filtration through pore sizes 0.1 µm and 0.22 µm had broken up the chromatin-like structures into smaller particles. (Fig. 2, lower panels).

**Fig. 3:**
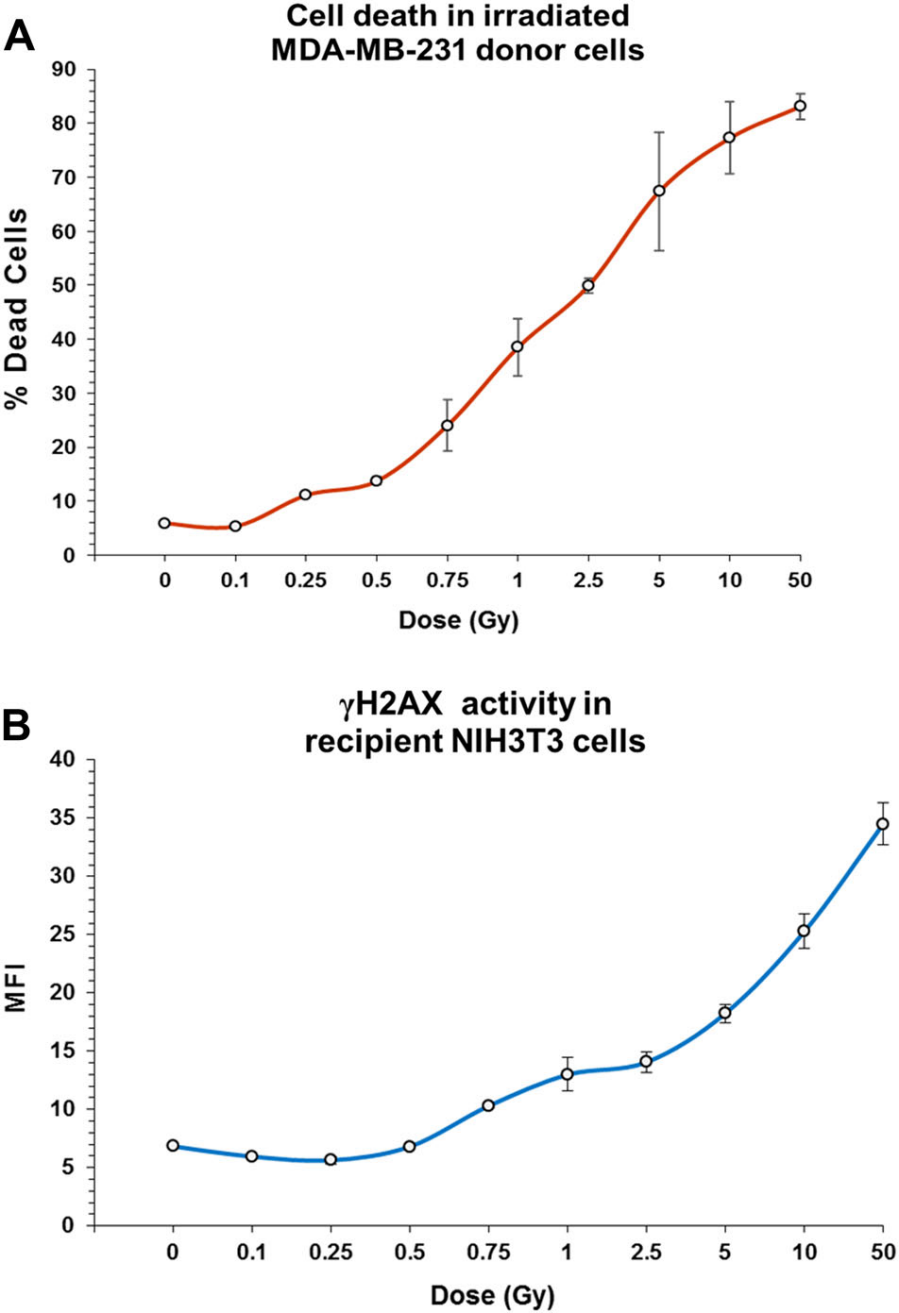
Dose-response relationship between degree of cell death in donor cells and bystander activation of H2AX in recipient cells. **A** Incremental increase in cell death in MDA-MB-231 donor cells with increasing dose of radiation. **B** Increasing bystander activation of H2AX in NIH3T3 recipient cells with increasing cell death in donor cells. The line curves depict the mean MFI (±S.E.); all experiments were done in duplicates

We next examine whether a dose-response relationship existed with respect to cfCh-induced RIBE. As a first step, we determined the proportion of cells that die after 6 h of incubation following serially increasing doses of radiation (0.1–50 Gy) to MDA-MB-231 human breast cancer cells by the acridine orange and propidium iodide staining method. Little cell death was observed at dose level of 0.1 Gy, but cell death increased thereafter in a dose-dependent manner reaching 83% at a dose of 50 Gy (Fig. 3A). Conditioned media from these serially irradiated MDA-MB-231 were filtered and applied to NIH3T3 mouse fibroblast cells. After 6 h of incubation, the treated cells were stained for γH2AX to examine the extent of induction of dsDNA breaks in the recipients. We detected gradually increase in activation of H2AX in a dose-dependent manner reaching a maximum at dose 50 Gy (Fig. 3B). We also found that vast majority of cell death (88%) following ionizing radiation was caused by apoptosis with the remainder (12%) being due to necrosis (Supplementary Fig. 4).

In subsequent studies, we pre-labeled NIH3T3 mouse fibroblast cells with BrdU, treated them with radiation (10-Gy) and filtered the conditioned medium through a 0.22 µm syringe filter and applied the filtrate to the same un-irradiated recipient cells. We clearly detected the presence of fine BrdU-labeled red fluorescent cfCh particles in the nuclei of the recipients when examined after 12 h of incubation (Supplementary Fig. 5A–D upper panels). Immunofluorescence staining (green) for γ-H2AX indicated evidence of DNA double-strand breaks in the BrdU containing bystander cells (Supplementary Fig. 5A upper panel). Concurrent treatment of the irradiated filtered conditioned medium with chromatin inactivating agents, namely, CNPs, DNase I, or R-Cu prior to applying them to recipient cells caused dramatic inhibition of BrdU and γ-H2AX fluorescence (Supplementary Fig. 5A second, third and fourth panels). These findings confirmed that BrdU-labeled cfCh particles that had been released from the irradiated cells were responsible for activation of H2AX in bystander cells. We also observed significant upregulation of the apoptosis marker active Caspase-3 (green) and inflammatory cytokines NFκB (green), and IL-6 (green) in the BrdU containing cells (Supplementary Fig. 5B–D upper panels). Concurrent treatment with CNPs, DNase I, or R-Cu again showed dramatic inhibition in the activation of all three markers (Supplementary Fig. 5B–D). These findings convincingly demonstrated that cfCh were also responsible for activation of apoptosis and inflammation in the un-irradiated bystander cells. When filtered conditioned medium of BrdU-labeled NIH3T3 cells that had not been irradiated were and applied to bystander cells, few BrdU signals were detectable and little activation of H2AX, active Caspase-3, NFκB, and IL-6 was seen signifying the absence of RIBE (Supplementary Fig. 6).

**Fig. 4:**
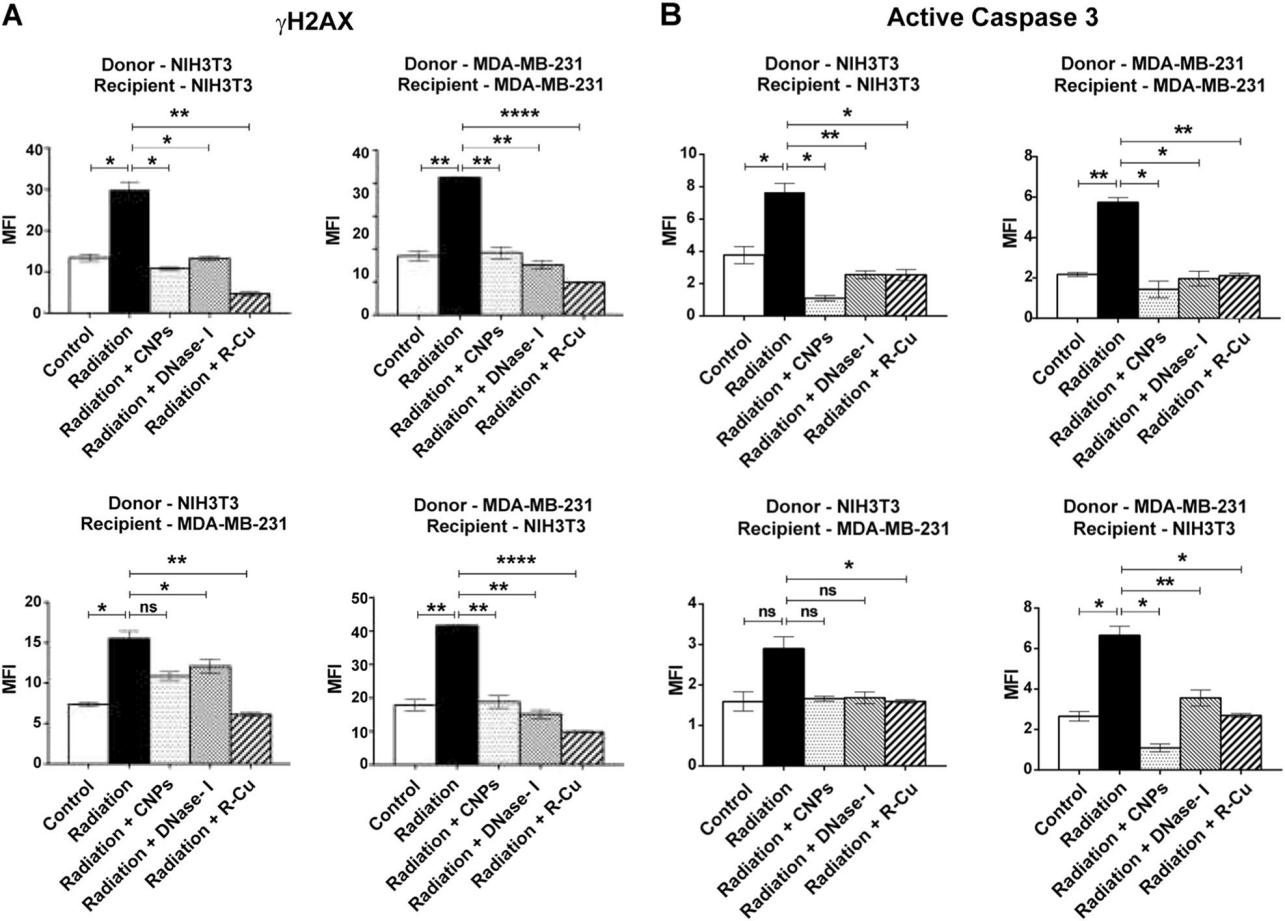

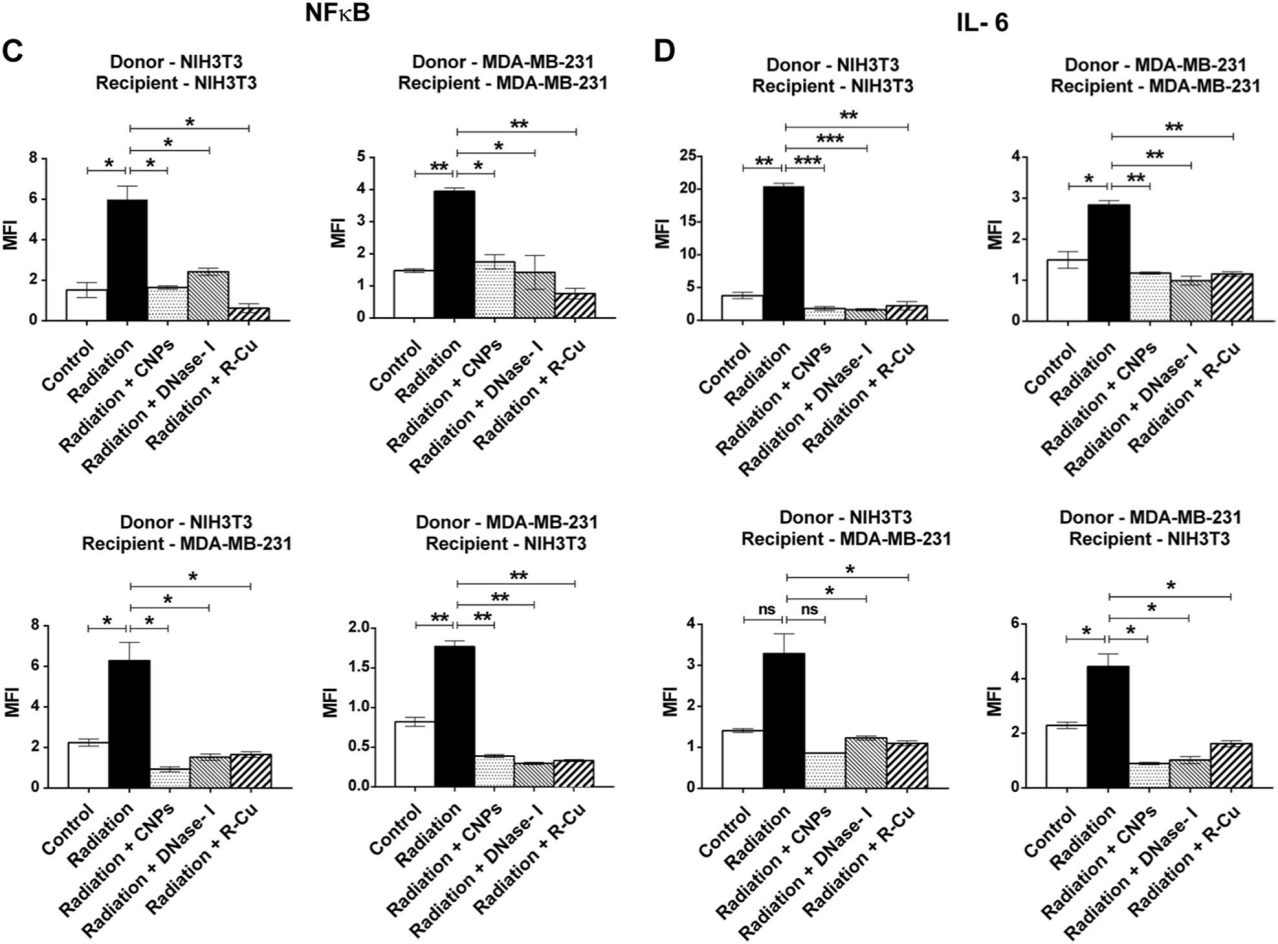
Filtered irradiated conditioned medium induce DNA damage, apoptosis, and inflammation in bystander cells. Various donor cells were irradiated (10 Gy) and after 6 h of incubation the culture media were passed through 0.22 µm filters. The filtered media were incubated with recipient cells for 6 h and analyzed for activation of RIBE. **A**–**D** The experiments were done in four different combinations of donor and recipient cell lines as depicted in the figure. Conditioned media from irradiated cells markedly activated H2AX, active Caspase-3, NFκB, and IL-6 in all cellular combinations. Pre-treatment of irradiated filtered conditioned medium with CNPs, DNase I and R-Cu completely prevented RIBE. The experiments were done in duplicate and the histograms depict mean MFI (±S.E.). The groups were compared using Student’s *t*-test. *p < 0.05; **p < 0.01, ***p < 0.001, NS Not significant

We next undertook a quantitative analysis of RIBE using four different donor–recipient combinations. The same experimental protocol as above was used except that the donor irradiated cells were not labeled with BrdU and the recipient cells were incubated with filtered media for 6 h . The various combinations examined were: (1) NIH3T3 (donor) and NIH3T3 (recipient); (2) MDA MB-231 (donor) and NIH3T3 (recipient); (3) MDA MB-231 (donor) and MDA MB-231 (recipient); (4) NIH3T3 (donor) and MDA MB-231 (recipient). We clearly observed statistically significant activation of H2AX, active Casapse-3, NFκB, and IL-6 in all 4 cellular combinations (Fig. 4A–D). Treatment with the three cfCh inactivating agents led to near-complete inhibition of all four RIBE parameters examined viz., inhibition of DNA double-strand breaks, apoptosis and inflammation. Inhibition was observed to a similar degree in all 4 combinations of donor and recipient cells (Fig. 4A–D). The observation that γ-H2AX, active Caspase-3, NFκB, and IL-6 could be inhibited by cfCh inactivating agents confirmed that cfCh were directly responsible for activation of RIBE. We demonstrate in control experiments that cfCh inactivating agents themselves had little effect in inducing RIBE parameters γ-H2AX and NFκB (Supplementary Fig. 7). We further show by using filters with pore size of 0.1 µm, that cfCh particles <0.1 µm were also active in inducing RIBE albeit to a slightly lesser degree than that obtained with 0.22 µm filters (Supplementary Fig. 8).

**Fig. 5:**
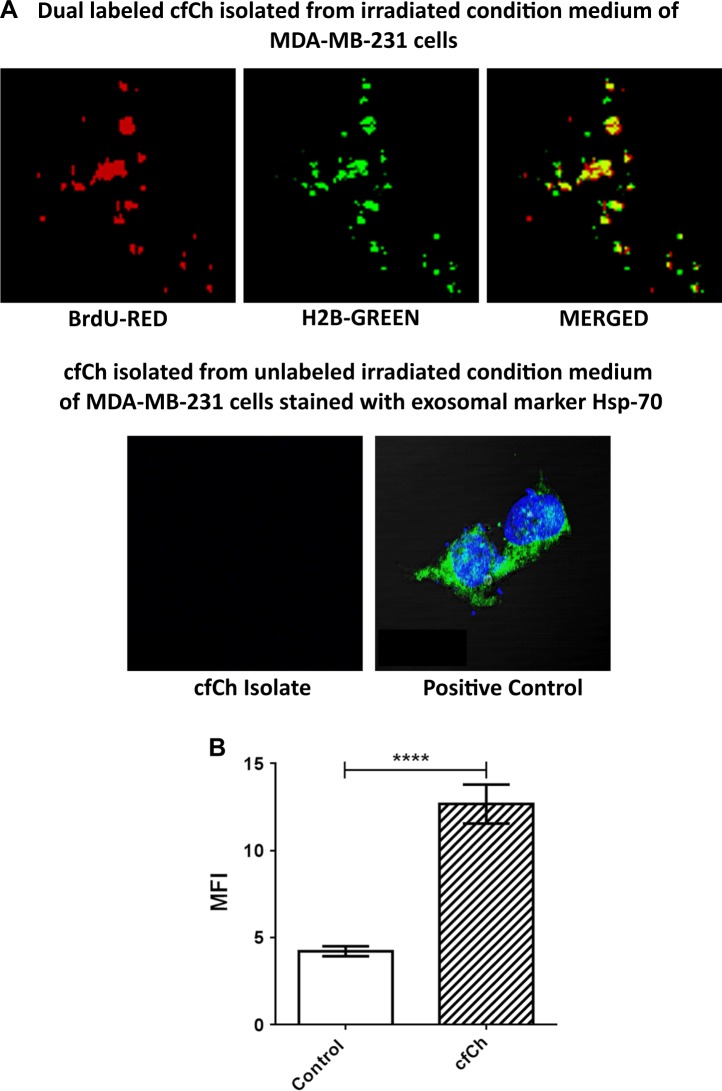
cfCh isolated from irradiated conditioned medium of MDA-MB-231 are devoid of exosomes but can activate H2AX in NIH3T3 cells. **A** Confocal images (zoomed ×60 magnification) of dually labeled cfCh isolates from irradiated conditioned medium of MDA-MB-231 cells (Upper panel). Confocal images of un-labeled cfCh isolates stained with exosome marker Hsp-70 showing absence of exosomes (lower left hand image). MDA-MB-231 cells stained with Hsp-70 are used as a positive control (lower right hand image). **B** Activation of H2AX in NIH3T3 cells by cfCh isolates from MDA-MB-231 cells that are devoid of exosomes (*p* = 0.0001).

As the irradiated conditioned media are known to contain exosomes^15,34^, we conducted experiments to investigate if exosomes might be responsible for the RIBE effects that we observed. To this end, we directly isolated cfCh from irradiated filtered conditioned medium of MDA-MB-231 cells that had been fluorescently dually pre-labeled in their DNA and histones and examined them under confocal microscopy. We clearly detected presence of dually labeled particles representing cfCh (Fig. 5A, upper panel). In a parallel set of experiments we isolated cfCh from conditioned medium from unlabeled MDA-MB-231 cells and stained the isolates with exosome markers Hsp-70. No staining with this marker was visible indicating that cfCh were free of exosomes (Fig. 5A, lower panel). Next, we applied cfCh isolates from 1 ml of irradiated conditioned medium to NIH3T3 cells and examined the cells for H2AX activation after 6 h of incubation. Fig. 5B clearly shows that cfCh isolates that are devoid of exosomes are potent activators of RIBE.

**Fig. 6:**
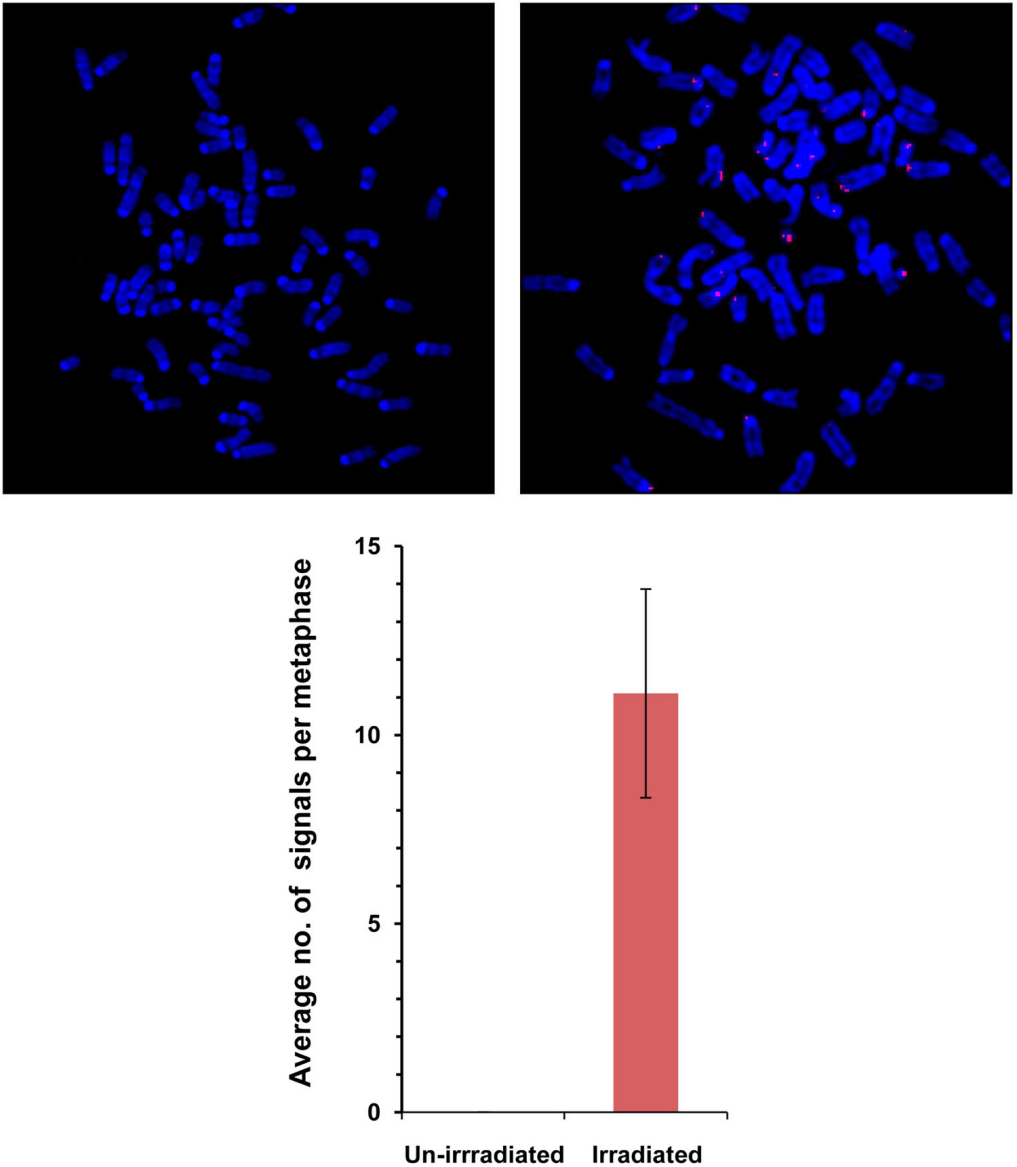
Detection of human DNA signals by FISH on chromosome arms of NIH3T3 mouse fibroblast cells. The donor MDA-MB-231 breast cancer cells were irradiated (10 Gy) and the filtered culture medium applied to NIH3T3 mouse fibroblast cells. FISH was performed using a human whole genomic probe at 10^th^ passage. Cells treated with conditioned medium of un-irradiated cells (left hand image); cells treated with conditioned medium of irradiated cells (right hand image). Quantitation of human signals is given in the lower panel (Mean ± S.E.). Thirty metaphases were analyzed in each case

**Fig. 7:**
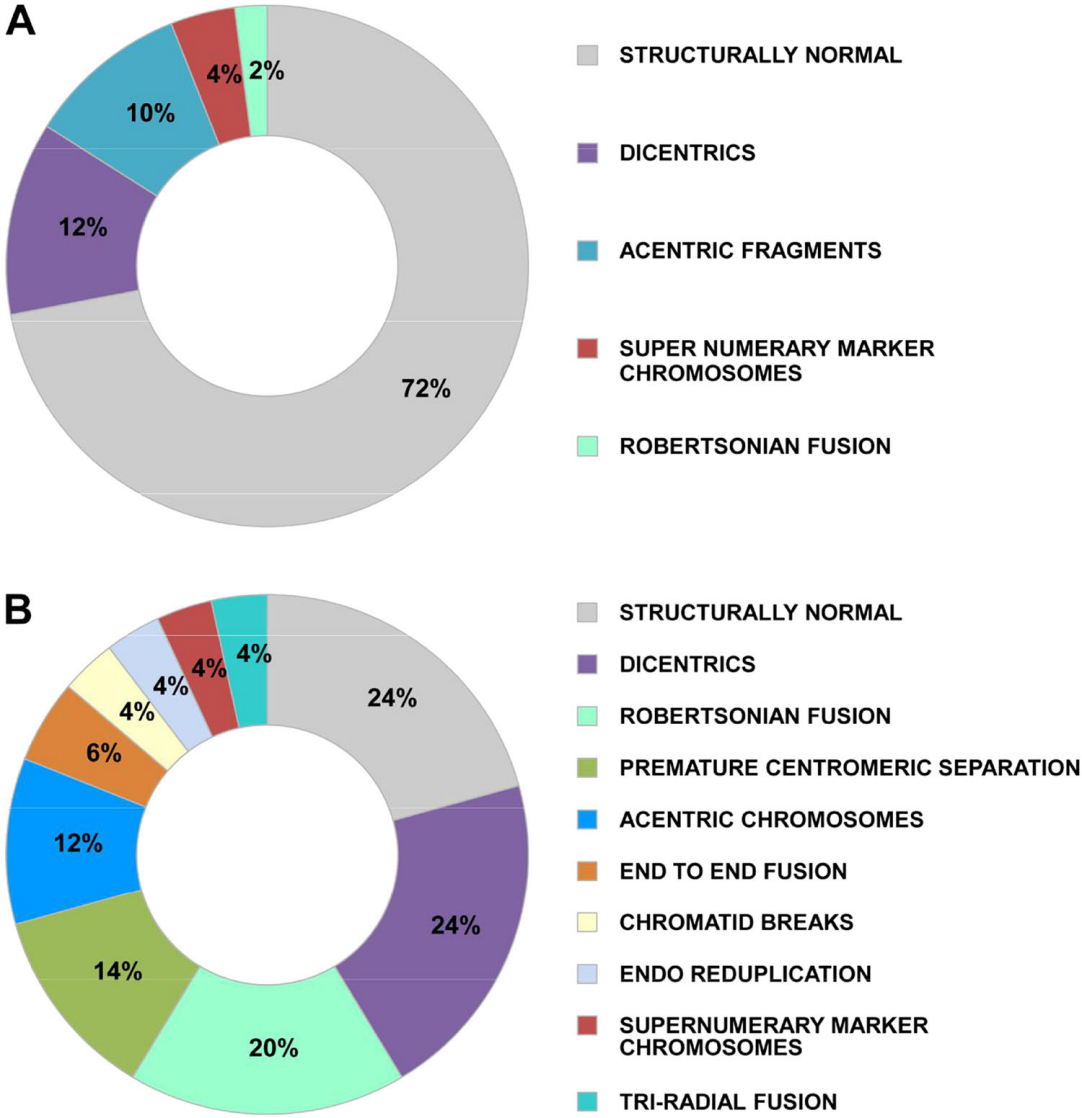
cfCh released into the conditioned medium of irradiated cells induce chromosomal aberrations in un-irradiated bystander cells. The experiment was done as described under Fig. 3 and karyotype analysis was performed at 10^th^ passage. **A** Wagon wheel depicting chromosomal aberrations in metaphase preparations from cells treated with conditioned medium of un-irradiated cells. **B** Wagon wheel depicting chromosomal aberrations in metaphase preparations from cells treated with conditioned medium of irradiated cells. The results depict those derived from analysis of 50 metaphase spreads

As we have earlier reported that circulating cfCh, or those released locally from dying cells, can integrate into chromosomes of healthy cells^23–25^, we investigated whether cfCh from irradiated cells would integrate into chromosomes of bystander cells. Conditioned medium from irradiated human breast cancer cells (MDA-MB-231) were filtered as above using 0.22 µm filters and applied to NIH3T3 cells. When metaphase spreads were prepared from the treated cells after 10 passages and examined by FISH for presence of human DNA signals using a human whole-genomic probe, numerous fluorescent human DNA signals were clearly detectable on chromosomal arms of the mouse cells (Fig. 6). No signals were detectable in cells treated with conditioned medium of un-irradiated cells. We next examined metaphase preparations from cells at 10^th^ passage to detect appearance of chromosomal aberrations as the latter is known to be a phenomenon associated with RIBE and is implicated in development of secondary cancers both at proximal and distant sites following primary irradiation^1^. Numerous chromosomal aberrations could be detected in mouse cells treated with conditioned medium of irradiated cells (Fig. 7). These included dicentrics, Roberstonian fusion, premature centromere separation, acentric chromosomes, end-to-end fusion, chromatin breaks, endo-reduplication, and tri-radial fusions (Supplementary Fig. 9). We found that 72% of cells treated with conditioned medium of un-irradiated cells had structurally normal chromosomes—a figure similar to that observed in untreated control NIH3T3 cells (data not shown). However, only 24% of cells treated with conditioned medium of irradiated cells showed structurally normal chromosomes (*p* < 0.0001) (Fig. 7). This data confirmed that cfCh are capable of inducing genomic damage and chromosomal instability following their integration into bystander cell genomes.

In order to investigate whether irradiated cells that survive, but carry damaged DNA extrude cfCh to induce RIBE? To address this issue, we irradiated MDA-MB-231 cells with a radiation dose of 0.1 Gy, which caused little cell death but had inflicted dsDNA breaks in 33.4% of cells (Fig. 3A and Supplementary Fig. 10, upper panel). Filtered conditioned medium from these cells, with extensive DNA damage, when applied to recipient NIH3T3 cells, however, did not lead to activation of RIBE (γH2AX) in the bystander cells. The level of H2AX activation was similar to that seen when un-irradiated conditioned medium had been applied to recipient NIH3T3 cells (Supplementary Fig. 10). These findings suggest that cfCh are released mainly from dying cells and not from those with dsDNA breaks without accompanying cell death.

We demonstrate that bystander effects can also occur when cells are exposed to chemotherapeutic agents by showing that Adriamycin treated cells also release cfCh into the culture medium. When conditioned media of Adriamycin treated cells were filtered and applied to NIH3T3 cells, a strong γH2AX response was observed (Supplementary Fig. 11).

**Fig. 8:**
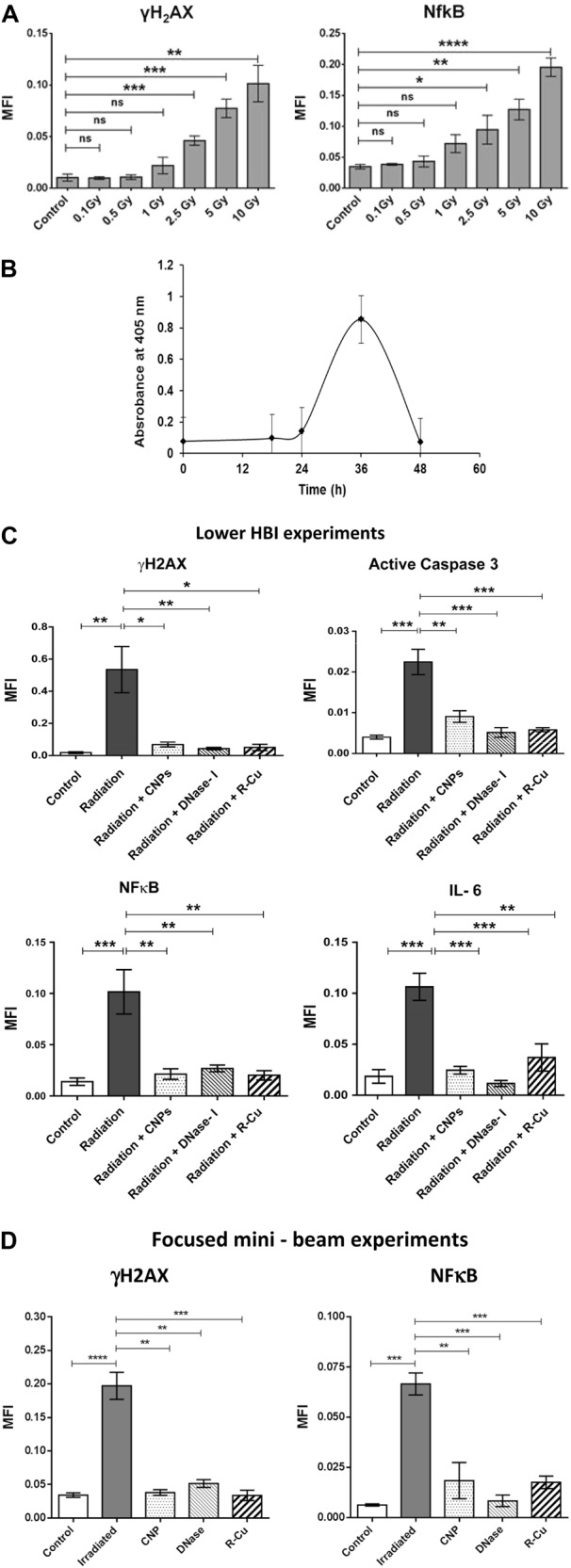
Activation of DNA damage and inflammation in brain cells of mice delivered lower hemi-body and focused mini-beam radiations and their abrogation by concurrent treatment with cfCh inactivating agents. **A** Dose-response effect of out-of-field RIBE following lower HBI in brain cells with respect to γH2AX and NFκB. **B** Graph showing surge of cfCh in circulation at 36 h following lower HBI measured by Cell Death Detection ELISA^plus^ kit wherein results are expressed in terms of absorbance kinetics at 405 nm. **C** Inhibition of out-of-field RIBE with respect to γH2AX, active Caspase -3, NFκB, and IL-6 by CNPs, DNase I and R-Cu. All animals except the control group received lower HBI (10 Gy) with and without CNPs, DNase I, and R-Cu. **D** Inhibition of out-of-field RIBE with respect to γH2AX and NFκB. All animals except the control group received focused min-beam radiation (20 Gy) with and without CNPs, DNase I, and R-Cu. The control and irradiated groups comprising five animals each while those receiving radiation plus CNPs, DNase I and R-Cu comprised of three animals each. All histograms depict mean MFI (±S.E.). The groups were compared using Student’s *t*-test. *p < 0.05; **p < 0.01, ***p < 0.001

In order to investigate whether cfCh are involved in out-of-field RIBE in an in vivo animal model, we irradiated the lower halves of bodies of mice with their upper halves being shielded from ionizing radiation by lead blocks (Supplementary Fig. 2). Serially increasing radiation doses used were 0.1 Gy, 0.5 Gy, 1 Gy, 2.5 Gy, 5 Gy, and 10 Gy. Dosimetry data for the various doses is given in Supplementary Table 2 wherein it is seen that brain received only 4–8% of the delivered dose. Estimation of γH2AX and NFκB foci showed activation of RIBE in brain cells which commenced from a dose of 1 Gy, although statistically significant differences were seen in doses of 2.5 Gy and above with a clear dose-response relationship being observed (Fig. 8A). To confirm that the RIBE effects seen in the brain were due to cfCh released into circulation from dying cells of the lower half of the body, we estimated circulating cfCh levels at various time points following lower HBI of 10 Gy. We observed a surge of cfCh into the circulation which reached a peak at 36 h post radiation (Fig. 8B). That RIBE was restricted to neuronal cells was confirmed by concurrent staining of brain cells with antibody against Glial Fibrillary Acidic Protein (GFAP) and that against γH2AX (Supplementary Fig. 12). It is clearly seen that nuclei showing γH2AX signals are surrounded by cytoplasmic staining for GFAP.

Confirmation that cfCh were involved in out-of-field RIBE seen above came from experiments, in which animals receiving 10 Gy lower HBI and were concurrently treated with cfCh inactivating agents namely, CNPs, DNase I, and R-Cu. As expected, 10 Gy lower HBI resulted in marked upregulation of γH2AX, active Caspase-3, NFκB, and IL-6 in brain cells. However, activation of these biomarkers were dramatically reduced by concurrent treatment with cfCh inactivating agents (*p* < 0.05–*p* < 0.001) (Fig. 8C and Supplementary Fig. 13A–D). The finding that biomarkers of RIBE could be inhibited by the three cfCh inactivating agents indicated that activation of γH2AX, active Caspase-3, NFκB, and IL-6 were unlikely to be due to radiation scatter. Rather, they indicated that agents responsible for out-of-field RIBE in brain cells were cfCh particles released into circulation from dying cells of animals treated with lower HBI.

In order to eliminate any element of doubt that the out-of-field bystander effects in brain cells in our HBI experiments might be due to radiation scatter, we irradiated mice using Varian/BrainLAB Novalis Tx™ mini beam radiotherapy platform. Mice were subjected for focused mini beam 2000 cGy dose over the umbilical region under general anesthesia; and they were killed after 48 h and brains were examined for activation of RIBE. Dosimetry revealed that the brain had received negligible radiation scatter (Supplementary Fig. 3). Immuno-flourescence analysis of brain showed significant activation of H2AX and NFκB which were markedly inhibited in mice which had been treated with the three cfCh inactivating agents (Fig. 8D).

## Discussion

We have recently reported that cfCh from dying cancer cells can integrate into genomes of bystander healthy cells to induce DNA damage and inflammation^23^. Herein we report results of multiple additional experiments and new evidence which strongly implicates cfCh in activation of RIBE. Results of new experiments reported herein include; (1) demonstration that, following ionizing radiation, cfCh are released predominantly from apoptotic rather than necrotic cells; (2) EM demonstration of presence of cfCh in conditioned media of irradiated cells; (3) demonstration that fluorescently labeled cfCh released from irradiated dying cells are freely ingested by bystander cells to localize in the nuclei by 6 h; (4) demonstration that cfCh released from irradiated cells integrate into genomes of bystander cells leading to extensive genomic instability; (5) demonstration that cfCh released from irradiated dying cells activate RIBE in the form of activation of H2AX, active Caspase-3, NFκB, and IL-6 in bystander cells; (6) demonstration that the above RIBE parameters can be abrogated by cfCh inactivating agents namely CNPs, DNase I and R-Cu; (7) demonstration of activation of RIBE parameters using multiple cellular combinations that include tumor to non-tumor and human to mouse cell lines; (8) demonstration of a direct relationship between radiation dose and cfCh-induced RIBE; (9) demonstration that cfCh-induced RIBE parameters can also be activated following chemotherapy; (10) isolation of cfCh from irradiated conditioned medium and demonstration that cfCh thus isolated can directly activate RIBE; (11) demonstration that cfCh-induced RIBE is not due to contaminating exosomes; (12) demonstration that cfCh can induce out-of-field RIBE in brain cells of mice following lower hemi-body irradiation; (13) demonstration that RIBE induced in brain cells is mediated via release of cfCh into the blood stream from irradiated dying cells; (14) demonstration of a dose-response relationship between radiation dose and activation of RIBE in brain cells; (15) demonstration that focused mini-beam radiation delivered to umbilical region of mice, that produces little scatter to the brain, can induce RIBE; (16) demonstration that RIBE in brain cells induced by both lower HBI and focused mini-beam irradiation can be prevented by cfCh inactivating agents.

It is generally assumed that as RIBE occurs when the conditioned medium from irradiated cells are filtered through 0.22 µm filters that the putative RIBE inducing damaging signals are soluble factors^3,26^. We demonstrate by EM that RIBE-inducing signaling agents are particulate in nature in the form of cfCh (Fig. 2). The latter can be isolated from conditioned medium of irradiated cells by ultra-centrifugation and affinity chromatography, and when they are examined by confocal microscopy, appear as discreet particles (Fig. 5A). Furthermore that the isolated cfCh particles when applied to bystander cells evoke a strong γH2AX signal (Fig. 5B). Although, inflammatory cytokines have been associated with RIBE, they have been considered to be secreted by macrophages, which are attracted by radiation-induced dead or damaged cells^35^. We show the direct involvement of cfCh, in the absence of macrophages, in activation of H2AX by bystander cells (Fig. 5B).

We demonstrate that radiation-induced cell death is predominantly due to apoptosis (88%) rather than by necrosis (12%) (Supplementary Fig. 4). By undertaking radiation dose-response experiments we show a linear increase in cell death in the donor MDA-MB-231 breast cancer cells with increasing dosage of ionizing radiation between a range of 0.25 and 50 Gy with the lowest dose (0.1 Gy) showing little cell death (Fig. 3, upper panel). When filtered conditioned medium from the above serially irradiated cells were applied to NIH3T3 mouse fibroblast cells, RIBE in the form of H2AX activation also showed a dose-response relationship with respect to increasing radiation dose beyond a dose level of 0.5 Gy (Fig. 3, lower panel). This dose-response relationship in bystander cells is not consistent with the report by Smilenov et al. who employed single-cell irradiation using alpha particles and reported a lack of dose-response effect^36^. Differences in experimental design and type of radiation used may explain the discrepancy with respect to our findings.

We demonstrate by FISH that radiation-induced cfCh that are taken up by bystander cells get integrated into their chromosomes (Fig. 6). Metaphase preparations of NIH3T3 cells that had been co-cultured with irradiated Jurkat cells showed multiple fluorescent signals of human DNA in the mouse cell chromosomes. The fact that they were detectable even after the bystander cells had gone through 10 passages confirmed that cfCh from irradiated cells had stably integrated into bystander cellular genomes.

By conventional cytogenetic analysis, we demonstrate that genomic integration of cfCh had induced extensive chromosomal aberrations in successive generations of bystander cells (Fig. 7 and Supplementary Fig. 9).

We demonstrate that cfCh-induced RIBE is not unique to ionizing radiation but can be activated with cfCh release by cells treated with chemotherapy (Supplementary Fig. 11). In this context, we have recently reported activation of chemotherapy-induced bystander phenomenon in vivo in mice^37^. We showed that toxicity of chemotherapy is primarily due to release of cfCh from the initial round of drug-induced cell death, and that this triggered a cascading effect causing exaggerated DNA damage, apoptotic and inflammatory responses in bystander healthy cells^37^.

We demonstrate that cfCh are also involved in out-of-field RIBE in vivo. Lower HBI in mice led to activation of DNA damage, apoptosis and inflammation in cells of the brain (Fig. 8C and Supplementary Fig. 13). The involvement of cfCh was substantiated by the fact that RIBE activation could be dramatically reduced to near baseline levels by concurrent treatment with the three cfCh inactivating agents (Fig. 8C and Supplementary Fig. 13). We show that lower HBI had resulted in a surge of cfCh in circulation suggesting that cfCh particles from dying cells of the lower hemi-body had reached the brain via the blood stream to induce RIBE (Fig. 8B). We observed a dose-response effect with respect to out-of-field RIBE in brain cells which increased progressively and was significantly higher than the baseline RIBE parameters (γH2AX and NFκB) beyond the dose level of 1.0 Gy (Fig. 8a). Lower dose levels (0.1 Gy, 0.5 Gy, and 1 Gy) did not significantly activate out-of-field RIBE. Our findings of a dose-response relationship is somewhat at variance with that reported by Ventura et al.^12^ who delivered high dose (~50 Gy) localized synchrotron-generated radiation to mouse skin and observed persistent systemic genotoxic and immune responses which they attributed to a scatter effect. Differences from our results can be attributed to this scatter radiation effect, which could have induced an adaptive response thereby influencing/modifying any bystander damage effect. The differences may also be attributed to differences in experimental design between the two studies.

In order to dispel any element of doubt that the out-of-field bystander effects in brain cells in our HBI experiments was due to radiation scatter, we irradiated mice using Varian/BrainLAB Novalis Tx™ mini-beam radiotherapy platform. Mice were subjected to focused mini-beam 2000 cGy radiation of 1 cm diameter over the umbilical region. Dosimetry revealed that radiation scatter to the brain was equivalent to background levels (Supplementary Fig. 3). Immuno-flourescence examination shown activation of H2AX and NFκB which were markedly inhibited in mice which had received the cfCh inactivating agents (Fig. 8d).

In conclusion, our results demonstrate that cfCh can induce RIBE both in vitro and in vivo. The fact that cfCh inactivating agents namely CNPs, DNase I, and R-Cu could virtually abolish RIBE suggests that cfCh are important inducers of RIBE. Prevention of RIBE has clinical relevance as RIBE has been implicated in induction of morbid systemic side-effects^2^ secondary tissue damage^38^ and secondary malignancies^39^ following radiotherapy for cancer.

## Electronic supplementary material

Supplementary Figures

Legends to Supplementary Figures

Supplementary Tables
