# Supplementary material for: Prevention of radiation-induced bystander effects by agents that inactivate cell-free chromatin released from irradiated dying cells
Source: Cell Death Dis. 2018 Nov 15;9(12):1142. doi: 10.1038/s41419-018-1181-x (PMC6238009; doi:10.1038/s41419-018-1181-x)
Supplement: Supplementary file 1 — Supplementary Figures [file 41419_2018_1181_MOESM1_ESM.doc]

**Supplementary Figure 1**


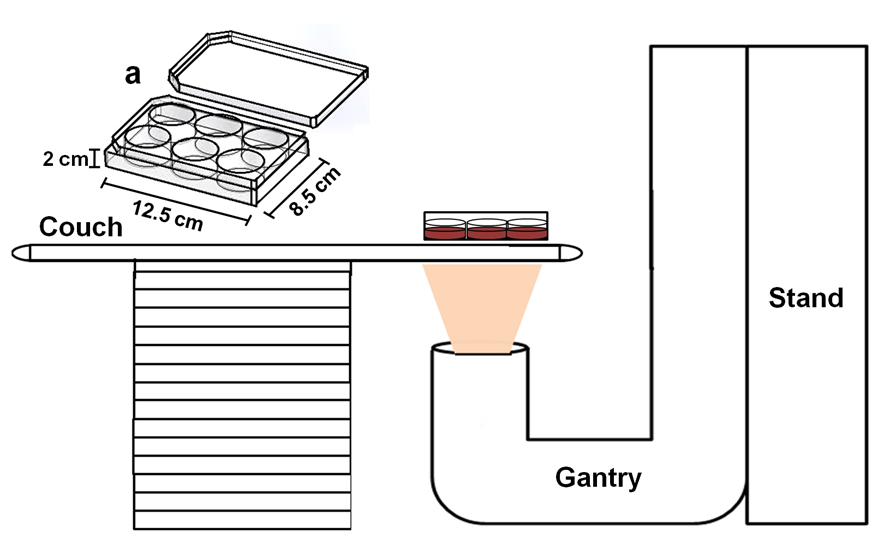


**Supplementary Figure 2**

**
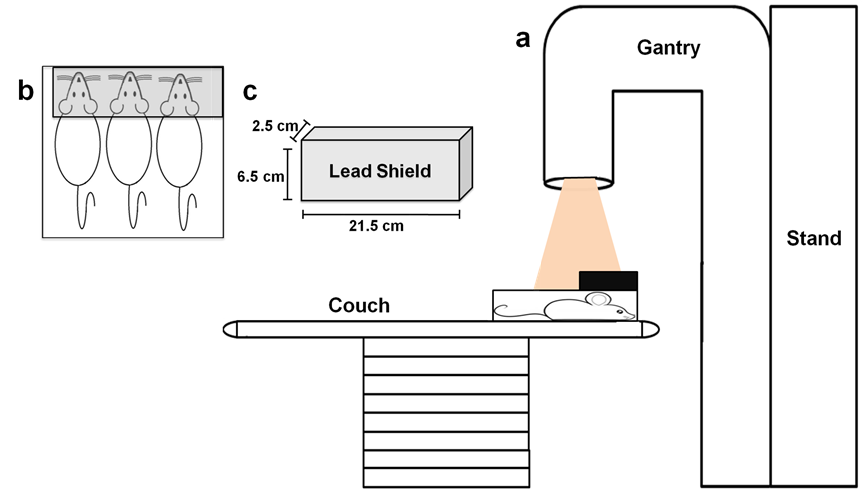
**

**Supplementary Figure 3**


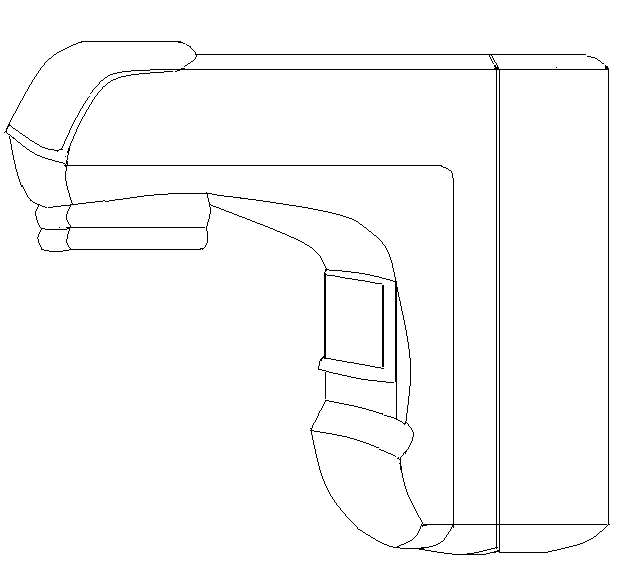

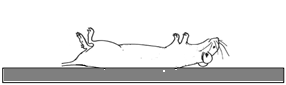


**A**


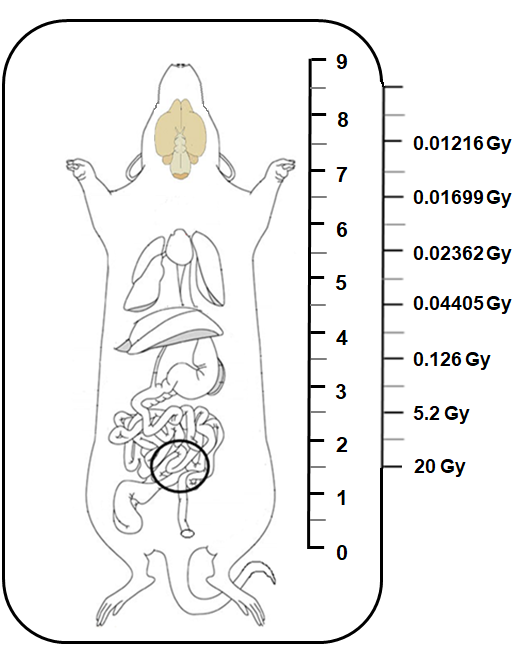


**B**

**Supplementary Figure 4**


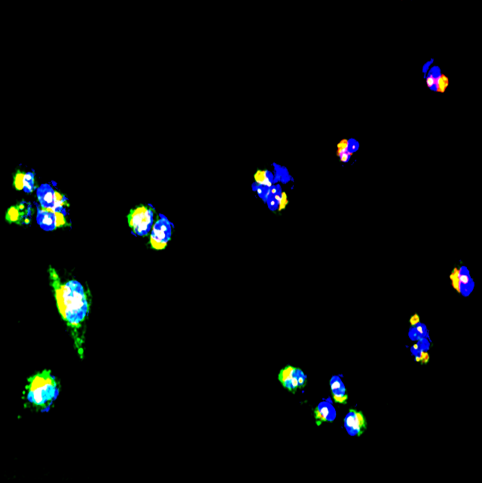

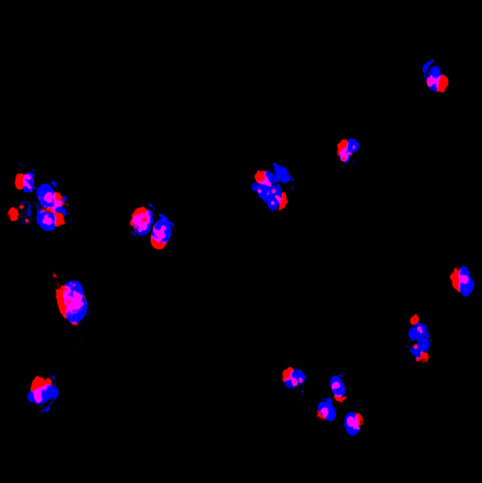

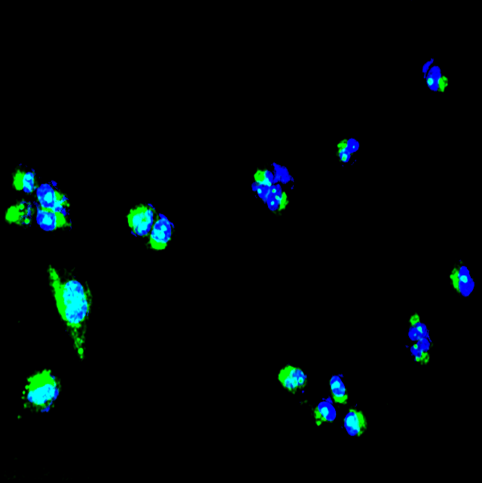


**CASPASE - 3 - GREEN**

**MERGED**

**CYCLOPHILLIN-A-GREEN**

**MERGED**


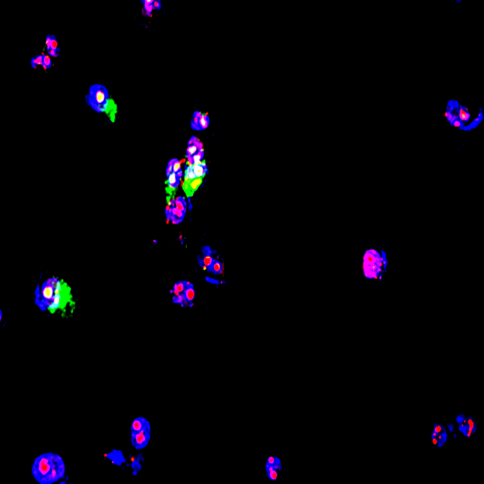

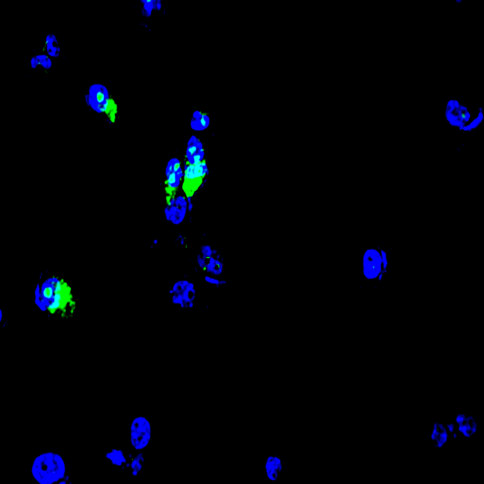

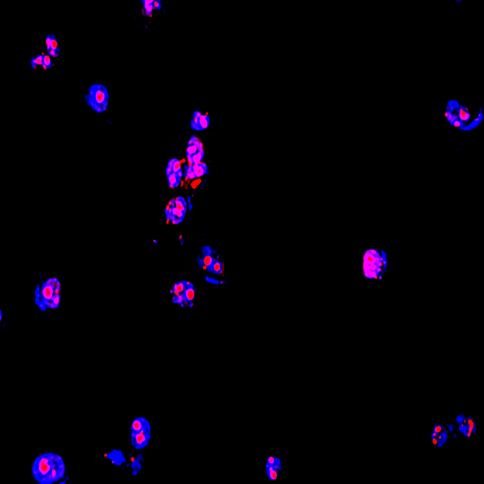


**PROPIDIUM IODIDE - RED**

**PROPIDIUM IODIDE - RED**

**Supplementary Fi 2:**

**A**

**Supplementary Figure 5**


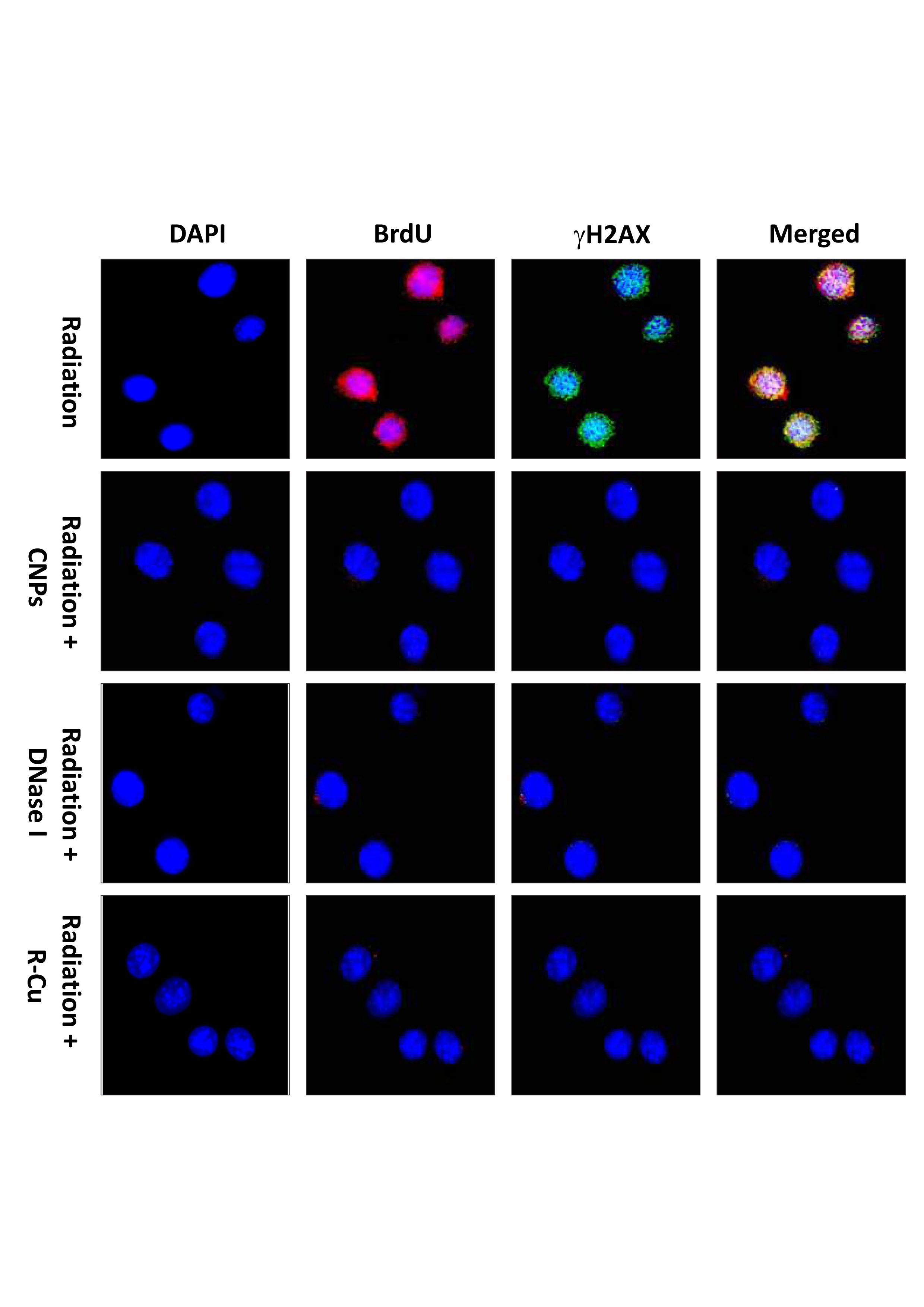
 **A**

**
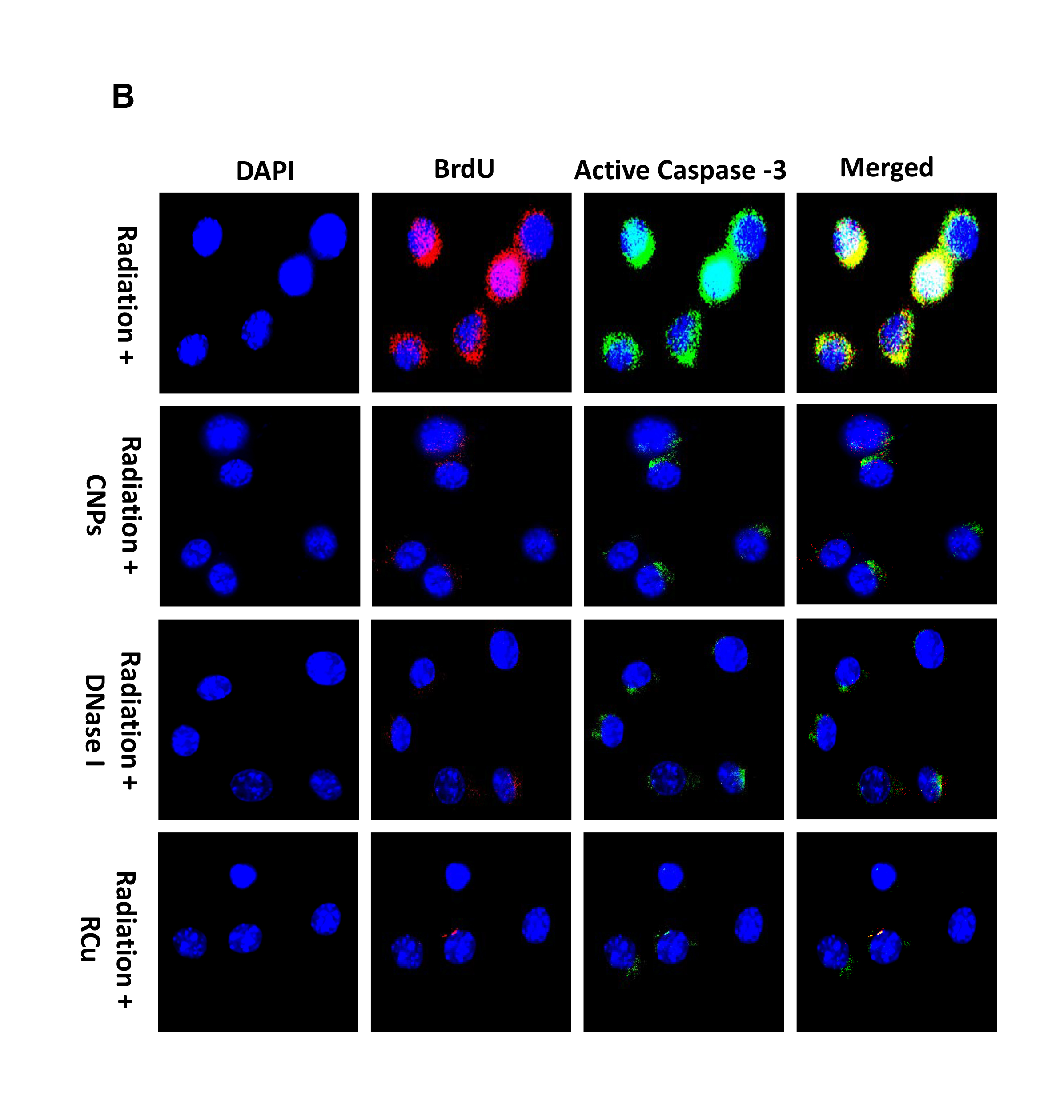
**


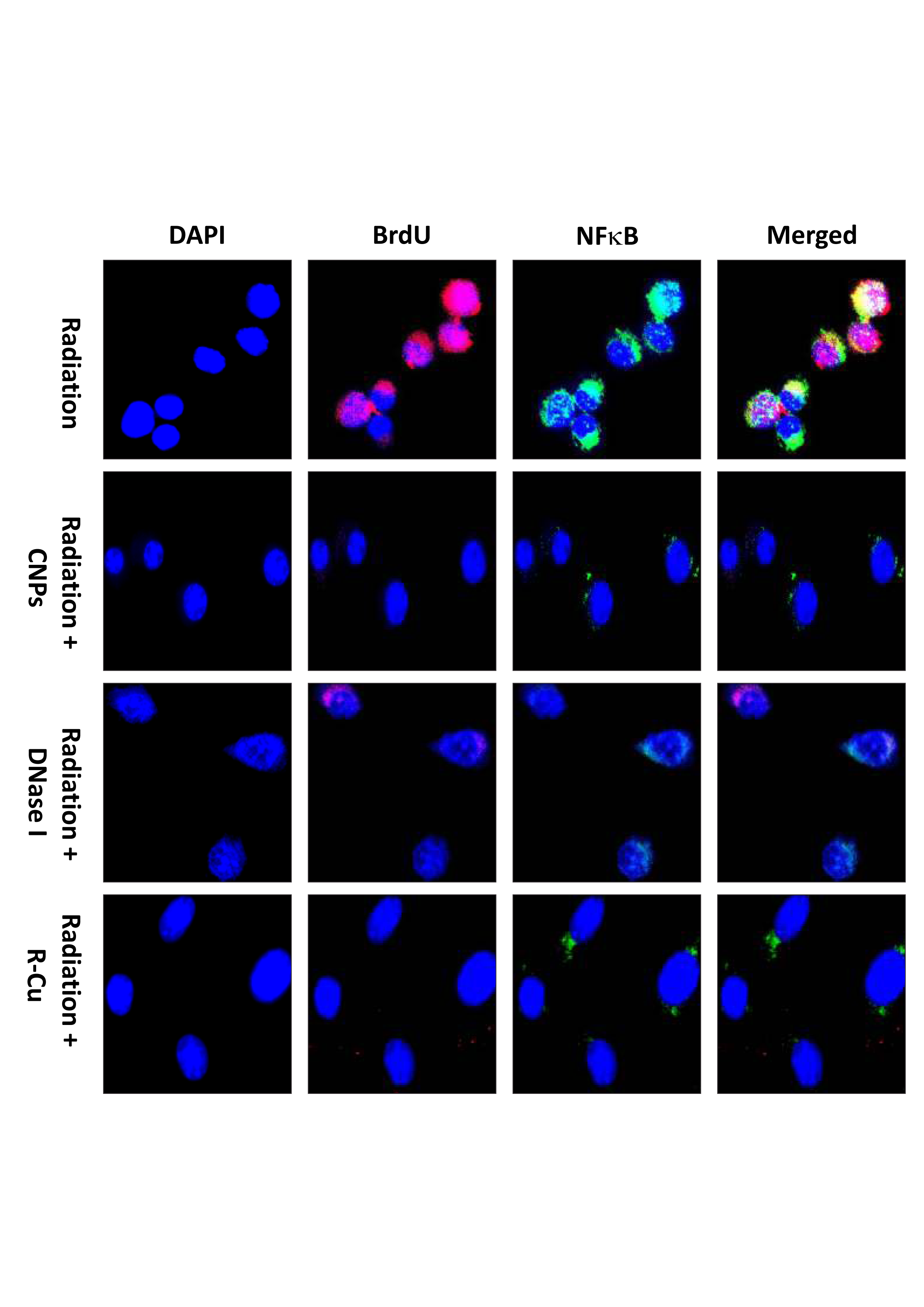
 **C**

**D**


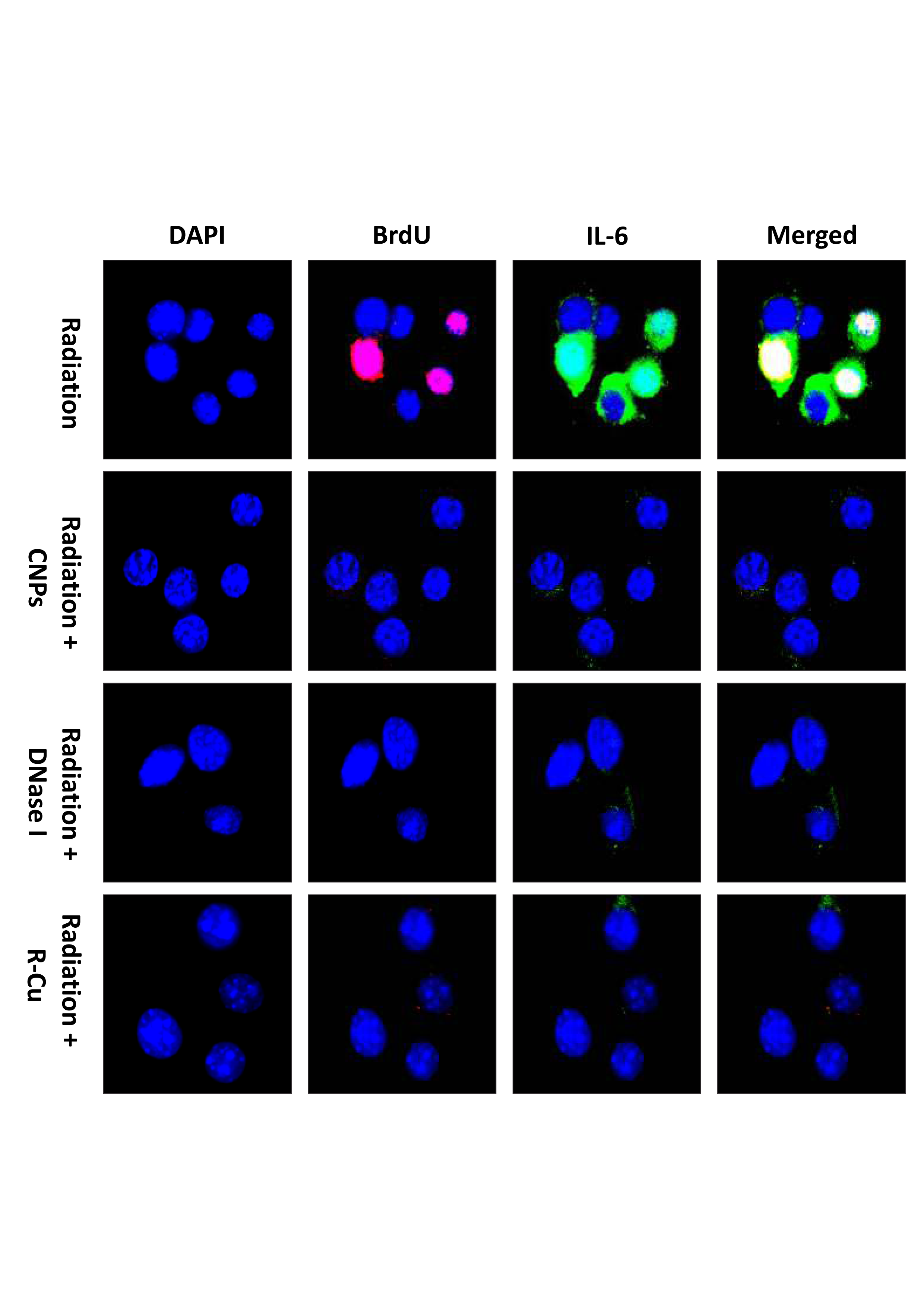


**Supplementary Figure 6:**

**
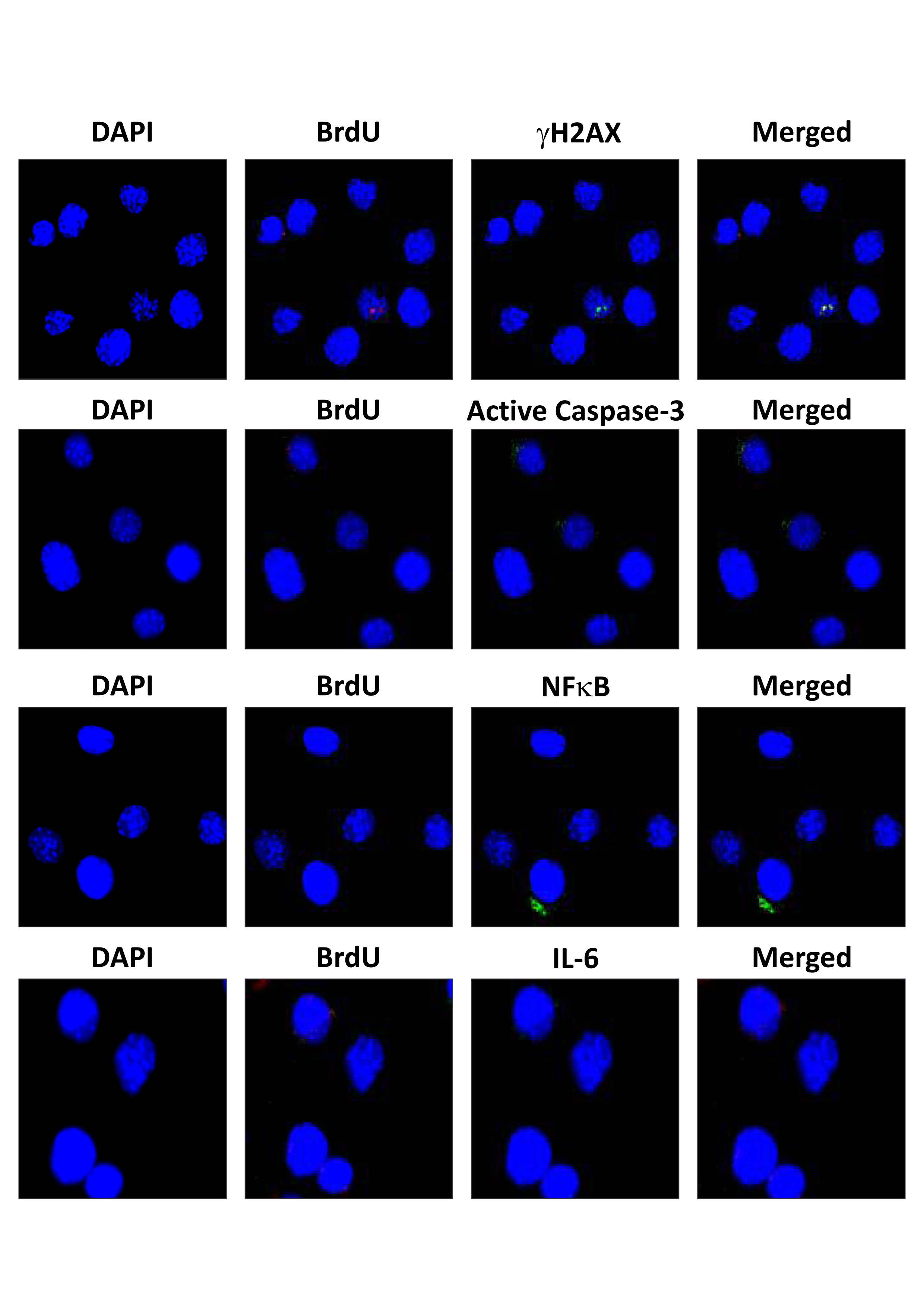
**

**Supplementary Figure 7:**

**
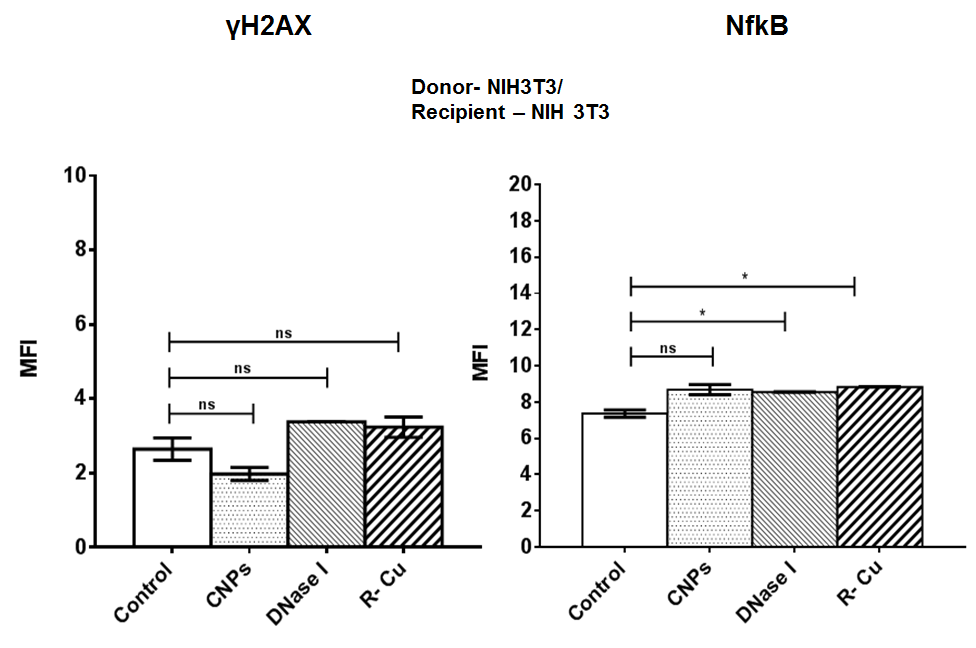
**

**Supplementary Figure 8:**

**
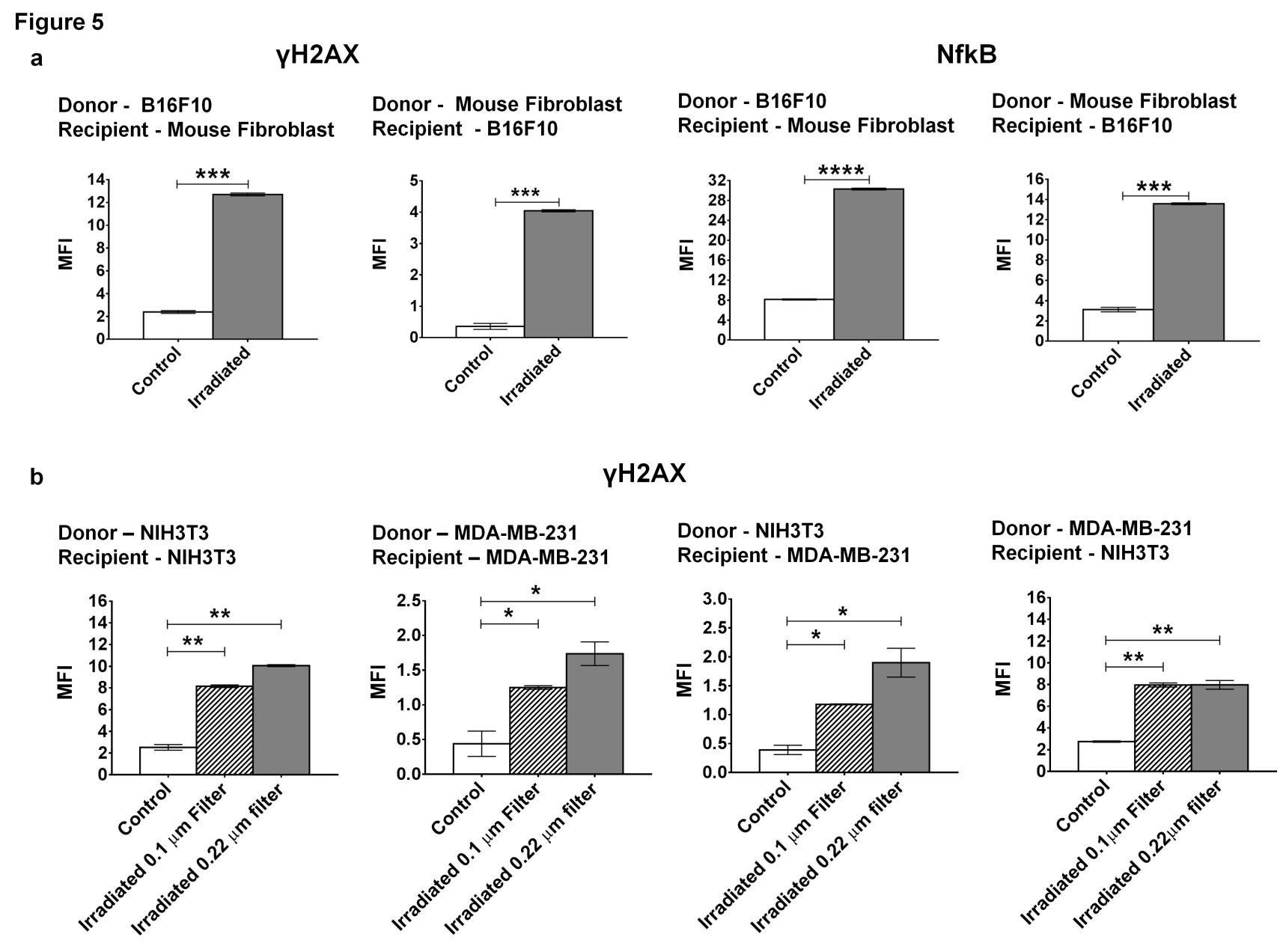
**

**Supplementary Figure 9:**

***
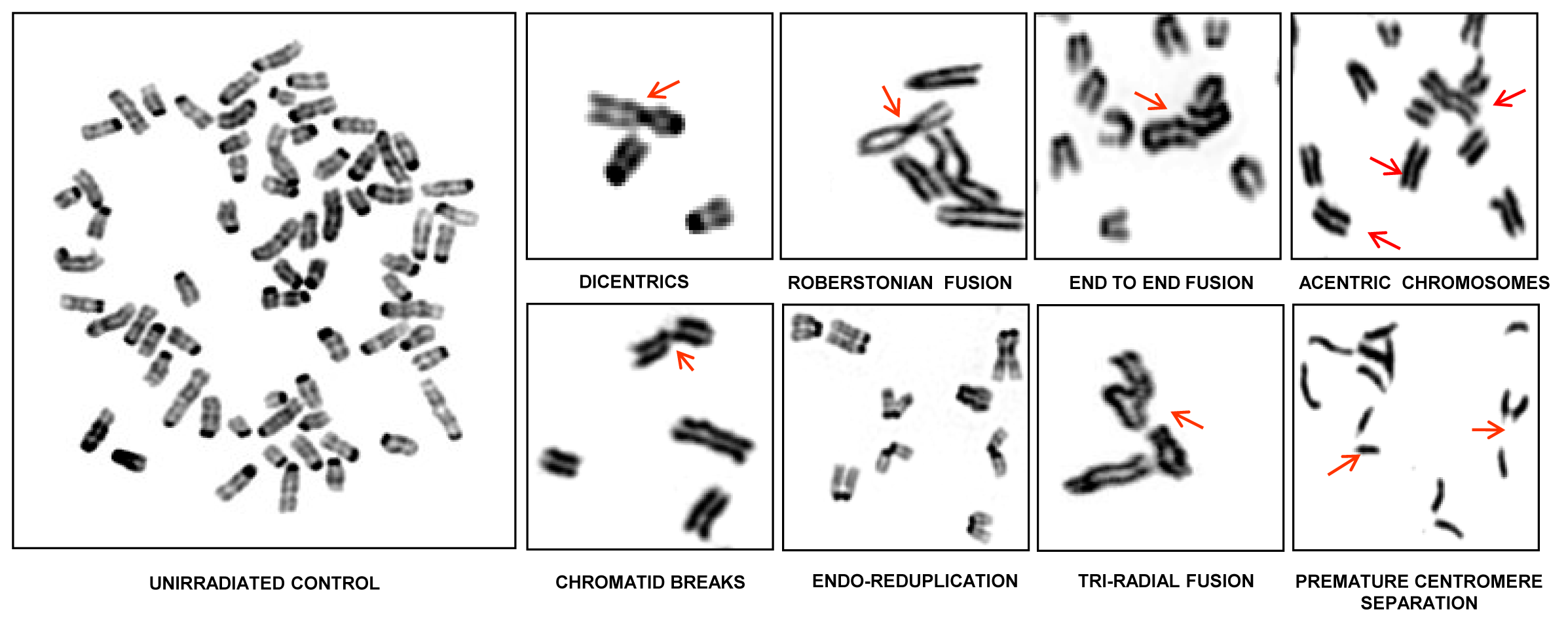
***

**Supplementary Figure 10:**

**
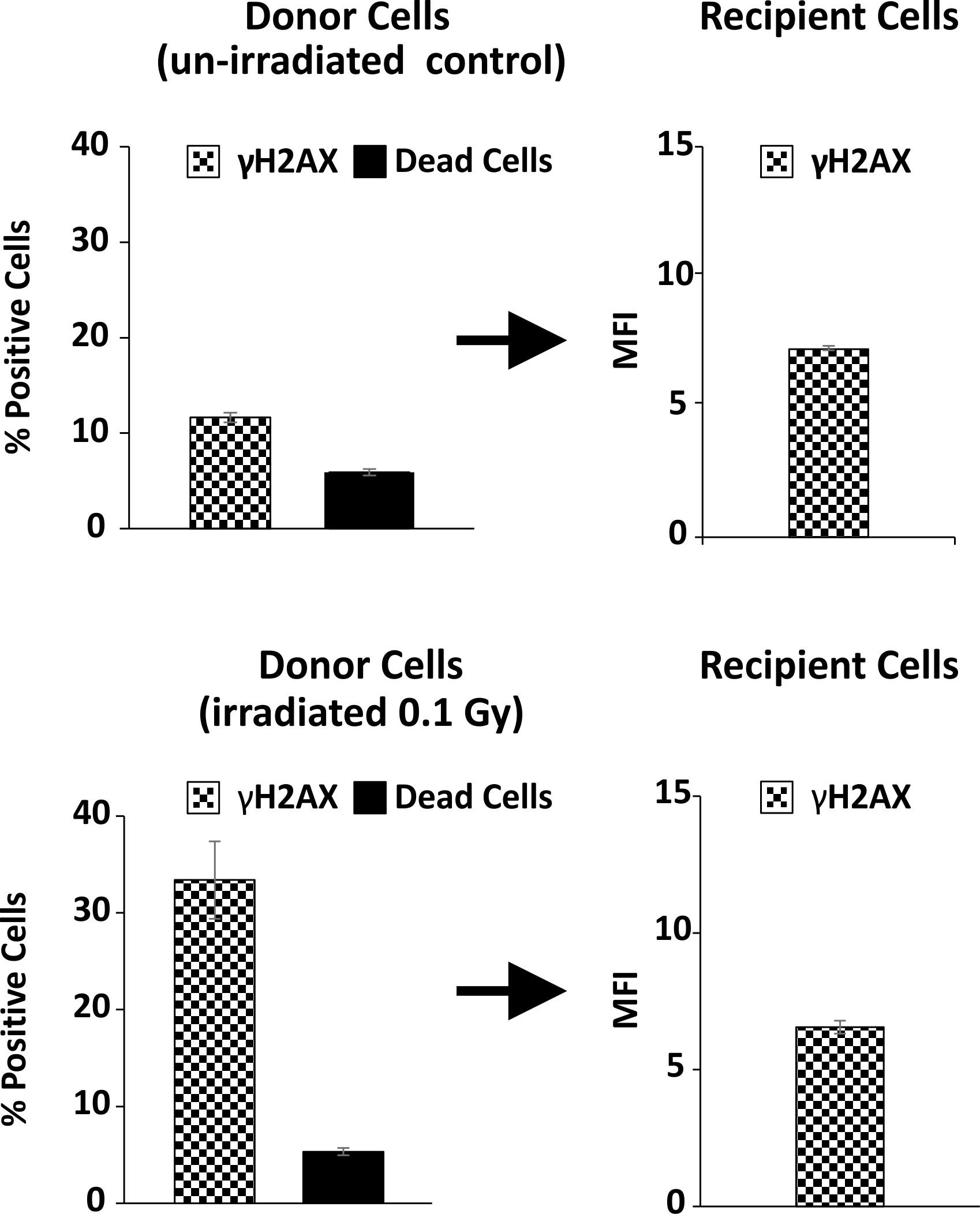
**

**
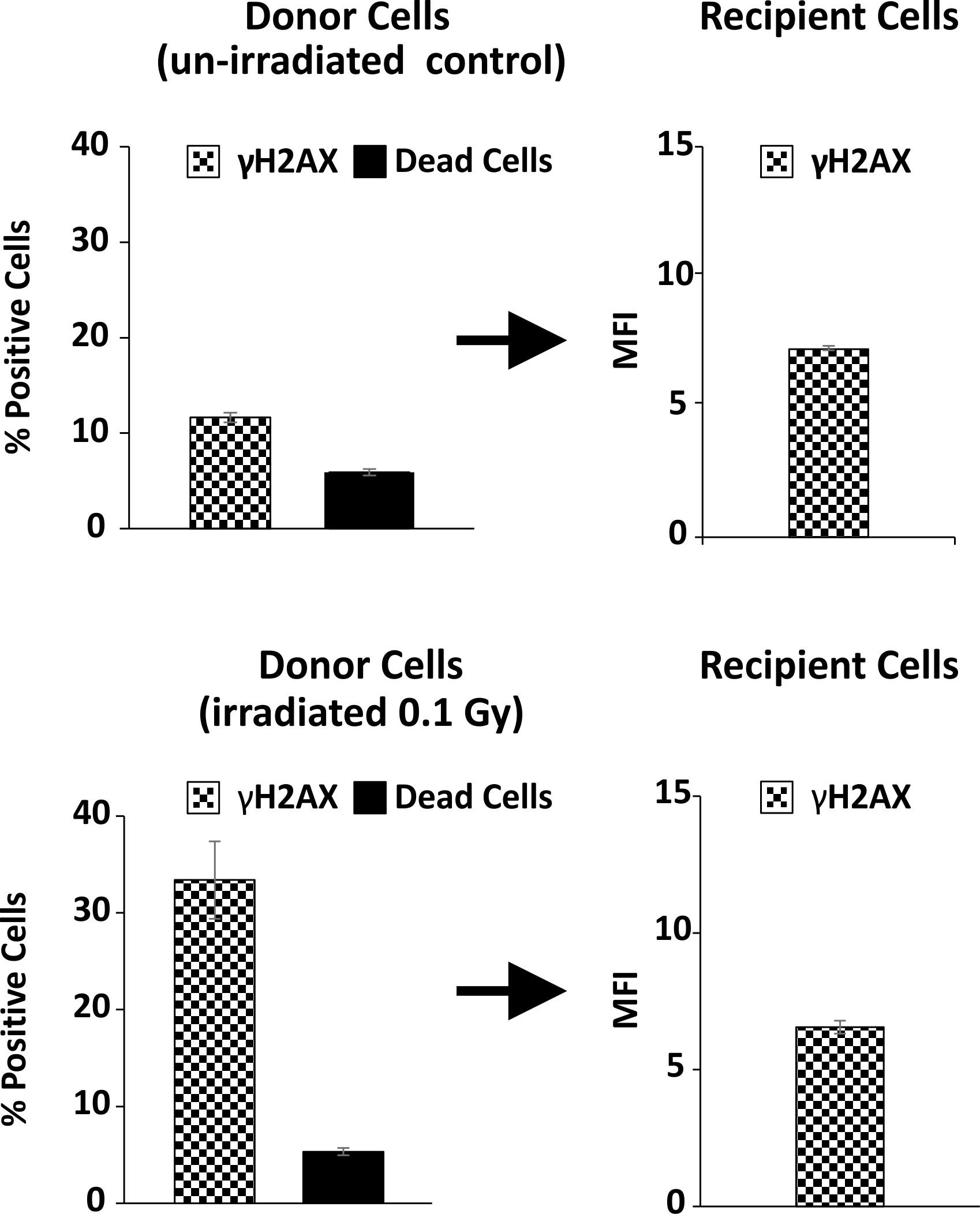
**

**Supplementary Figure 11:**

**
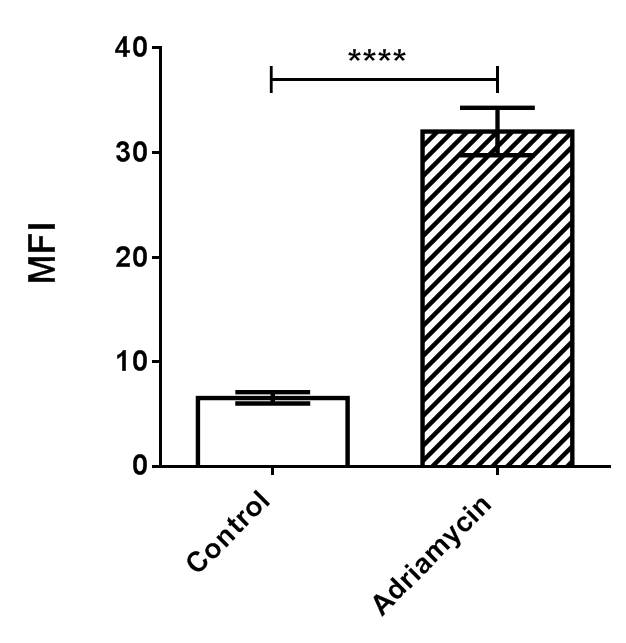
**

**Supplementary Figure 12**

**A**

**B**


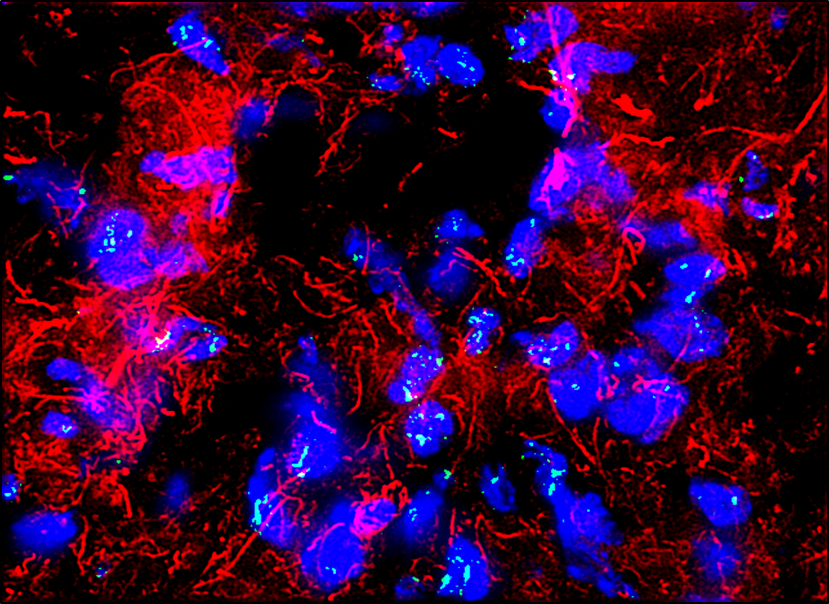

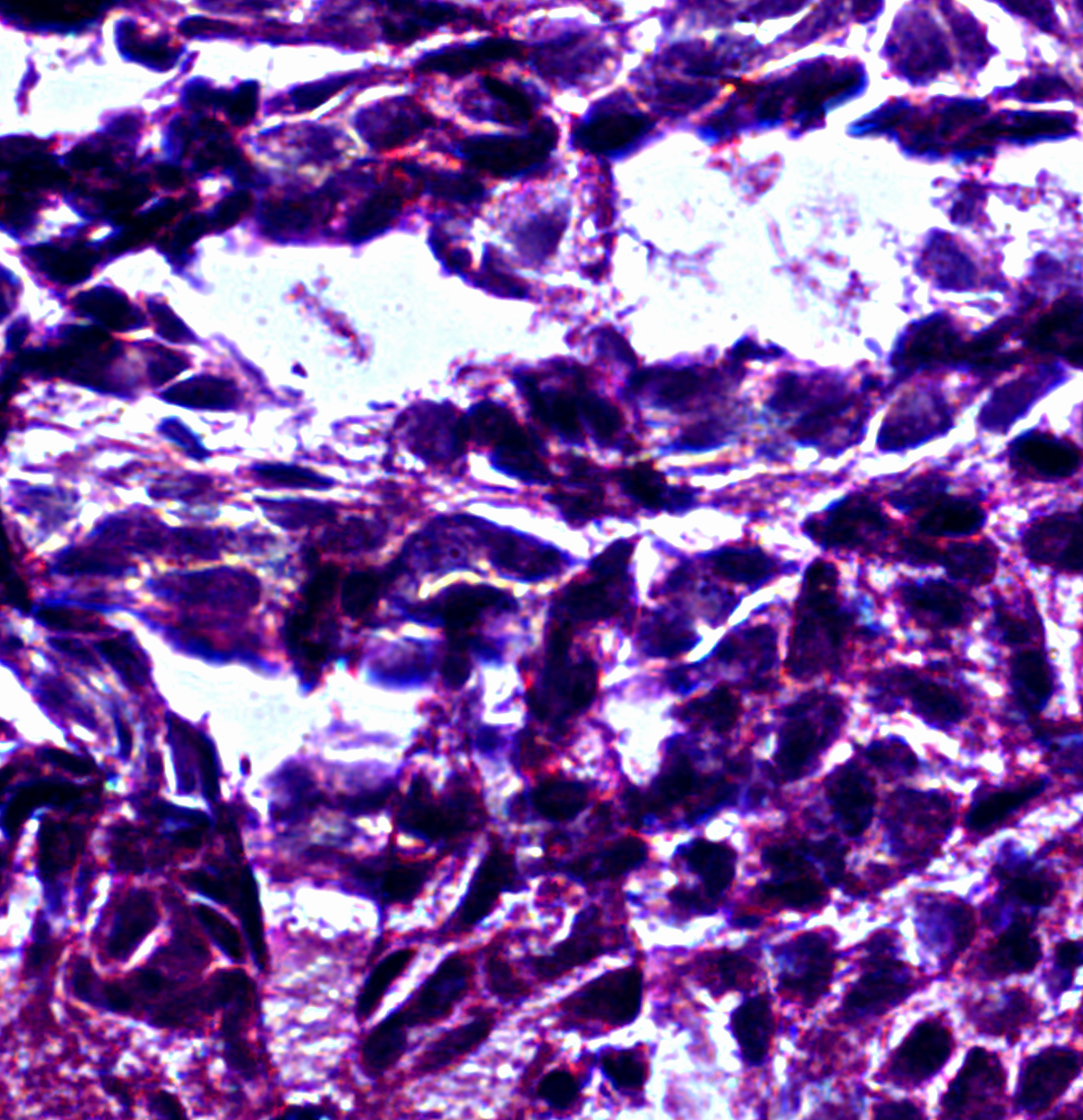


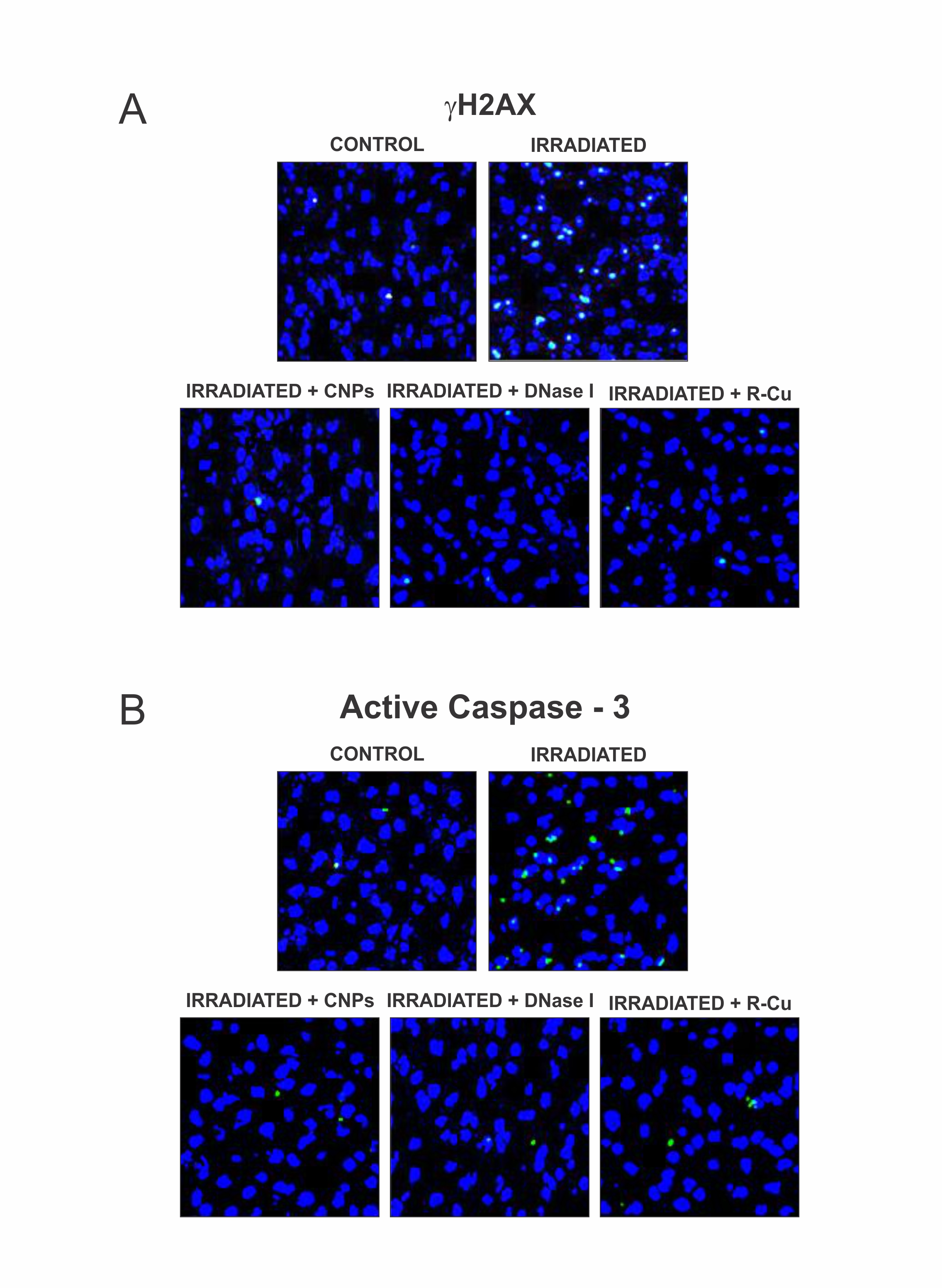
**Supplementary Figure 13:**

**
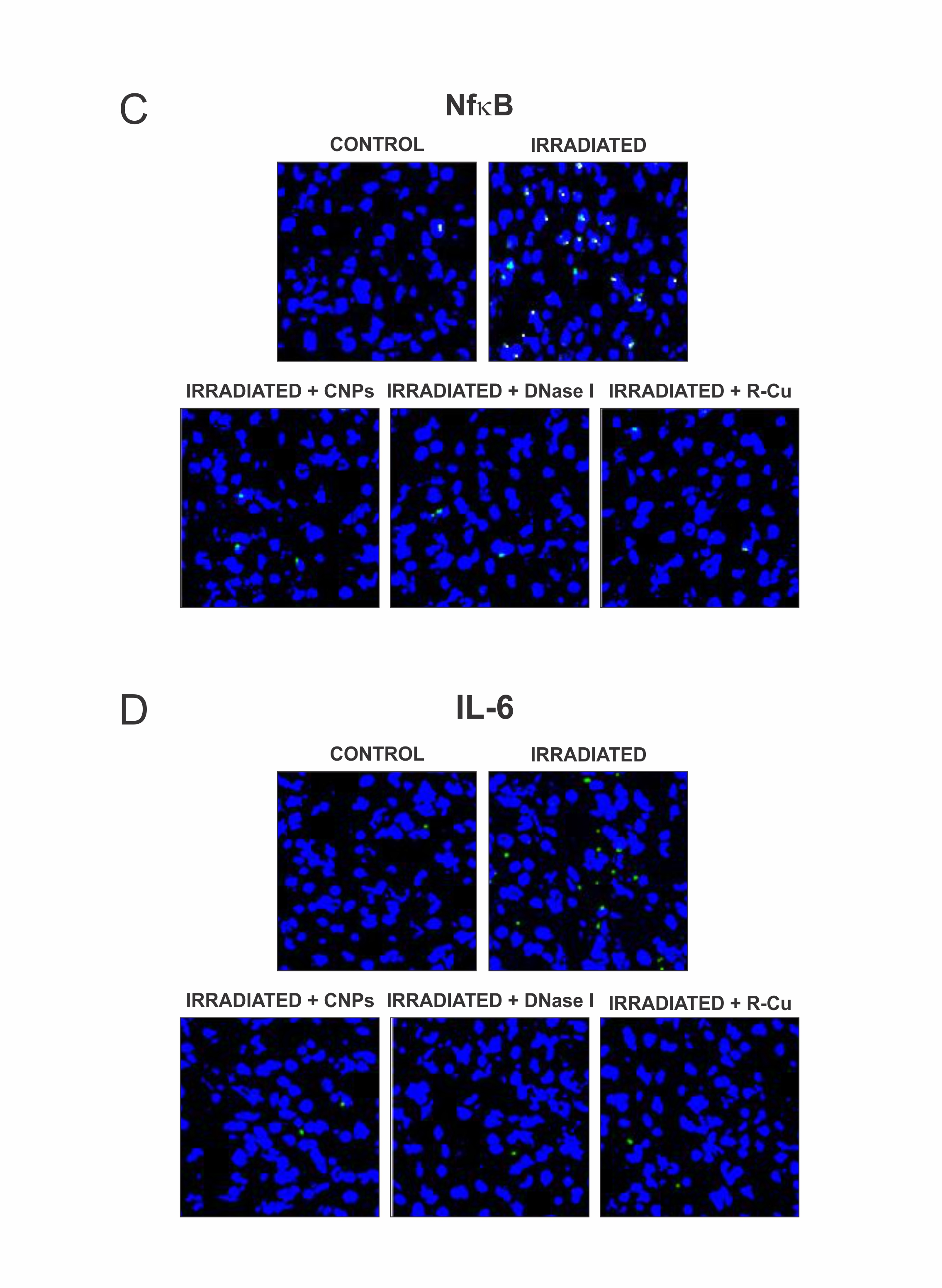
**
