# Supplementary material for: Prevention of radiation-induced bystander effects by agents that inactivate cell-free chromatin released from irradiated dying cells
Source: Cell Death Dis. 2018 Nov 15;9(12):1142. doi: 10.1038/s41419-018-1181-x (PMC6238009; doi:10.1038/s41419-018-1181-x)
Supplement: Supplementary file 2 — Legends to Supplementary Figures [file 41419_2018_1181_MOESM2_ESM.doc]

**Legends to Supplementary figures:**

**Supplementary figure 1:**

Schematic representation of experimental set-up for delivering radiation to cultured cells.

**Supplementary figure 2:**

Schematic representation of experimental set-up for delivering hemi-body radiation to mice using a telecobalt machine (Bhabatron-II)©.

**Supplementary figure 3:**

Schematic representation of experimental set-up for delivering focused mini-beam irradiation to mice. **A**. mouse dose delivery using Novalis Tx™ accelerator with HD120 MLC multileaf collimator for high resolution beam shaping. **B.** scatter radiation dose range from target to non-target regions.

**Supplementary figure 4:**

Representative fluorescent images of PI stained nuclei that co-localize with active Caspase – 3 (upper panel) and those that co-localize with cyclophilin-A (lower panel) for estimation of cell death due to apoptosis and necrosis respectively.

**Supplementary figure 5:**

Representative fluorescent microscopy images showing that BrdU labeled cfCh can pass through 0.22µm filters and enter into nuclei of recipient cells to induce DNA damage, apoptosis and inflammation. BrdU pre-labeled (red) donor cells were irradiated (10 Gy) and filtered after 6hr of incubation. The filtered condition media were incubated with recipient NIH3T3 cells for 12hr and analyzed for activation of various RIBE biomarkers. **A.** H2AX (green); **B.** active Caspase-3 (green); **C.** NFκB (green); **D.** IL-6 (green). Pre-treatment of irradiated conditioned medium with CNPs, DNase I and R-Cu completely prevented all four RIBE biomarkers. NIH3T3 cells were used as donors and un-irradiated NIH3T3 cells were used as recipients.

**Supplementary figure 6:**

Representative fluorescent microscopy images showing that treatment of recipient cells with filtered conditioned medium from un-irradiated BrdU-labeled cells show few BrdU signals in the recipient cells and little activation of H2AX, active Caspase-3, NFκB and IL-6. The experiments were done as in Supplemenatry figure 1 except that the donor cells were not irradiated.

**Supplementary figure 7:**

Experiments to demonstrate that cfCh inactivating agents by themselves have little effect on activation on DNA damage (γH2AX) and inflammation (NFκB). NIH3T3 cells were used for this study and the cfCh inactivating agents were added to the culture media, incubated for 6 hours at 37^o^C, filtered through 0.22µm filters and incubated with recipient NIH3T3 cells for 12 hours. Cells were analyzed for activation of H2AX and NFκB by immunofluorescence.

**Supplementary figure 8:**

Experiments to demonstrate that cfCh particles filtered through 0.1µm filters are also capable of activating H2AX in various combinations of cancerous and non-cancerous cells. The experiments were conducted as described in legends to Figure 4.

**Supplementary figure 9:**

Various forms of chromosomal aberrations detected in recipient NIH3T3 cells following treatment with conditioned medium from irradiated human breast cancer cells.

**Supplementary figure 10:**

Experiments to demonstrate that irradiated cells that survive but carry damaged DNA do not extrude cfCh to activate RIBE. The level of baseline γH2AX and percent dead cells in un-irradiated donor cells (MDA-MB-231) are shown in upper left hand histogram. The level of H2AX activation in recipient cells (NIH3T3) when treated with the above un-irradiated donor cells is shown in upper right hand histogram. Activation of H2AX in donor cells treated with 0.1 Gy which induced little cell death is shown in lower left hand histogram. Little H2AX activation is seen when condition medium of these donor cells were applied to recipient cells.

**Supplementary figure 11:**

cfCh from Adriamycin – induced cell death can induce RIBE. MDA-MB-231 cells were treated with Adriamycin (5µg/ml for 24 hr). Cells were trypsinised and extensively washed in PBS and incubated in fresh medium for 12 hr. The condition medium was passed through 0.22 µm filter and applied to NIH3T3 cells for 6 hr. Activation of H2AX was estimated by immuno-flourescence.

**Supplementary figure 12:**

Confirmation that RIBE is restricted to neuronal cells. **A**. Dual immunofluorescence using antibodies against Glial Fibrillary Acidic Protein (GFAP) and γH2AX to show that nuclei containing γH2AX signals are surrounded by cytoplasmic staining for GFAP. **B.** A consecutive section of the brain stained with Hematoxylin and Eosin. Magnification x 60.

**Supplementary figure 13:**

Representative fluorescent microscopy images of brain of mice treated with lower HBI showing activation of H2AX (**A**), active Caspase-3 (**B**), NFκB (**C**) and IL-6 (**D**) and their inhibition by concurrent treatment with cfCh neutralizing / degrading agents *viz*., CNPs, DNase I and R-Cu.
