# Supplementary material for: Prevention of radiation-induced bystander effects by agents that inactivate cell-free chromatin released from irradiated dying cells
Source: Cell Death Dis. 2018 Nov 15;9(12):1142. doi: 10.1038/s41419-018-1181-x (PMC6238009; doi:10.1038/s41419-018-1181-x)
Supplement: Supplementary file 3 — Supplementary Tables [file 41419_2018_1181_MOESM3_ESM.doc]

**Supplementary table 1:**

**List of antibodies** used

| Sr. No | Antibody | Source | Catalogue No. |
| --- | --- | --- | --- |
| 1 | anti-BrdU primary antibody | Abcam, USA | ab 6326 |
| 2 | Dylight-549 labeled anti-BrdU secondary antibody | Abcam, UK | ab98387 |
| 3 | γ-H2AX primary antibody | Abcam | ab26350 |
| 4 | active Caspase-3 primary antibody | Abcam | ab2302 |
| 5 | IL-6  primary antibody | Abcam | ab9324 |
| 6 | NFκB (p65)  primary antibody | Abcam | ab7970 |
| 7 | GFAP primary antibody | Dako, Glostrup Denmark | Z0334 |
| 8 | FITC labeled rabbit anti-mouse polyclonal antibody (γ-H2AX secondary antibody) | Chemicon International | AP160F |
| 9 | FITC labeled goat anti-rabbit polyclonal antibody (active Caspase-3 secondary antibody) | Chemicon International | AP307F |
| 10 | FITC labeled goat anti-rabbit polyclonal antibody (NFκB secondary antibody) | Chemicon International | AP307F |
| 11 | FITC labeled rabbit anti-mouse polyclonal antibody (IL-6 secondary antibody) | Chemicon International | AP160F |
| 12 | TRITC labeled donkey anti-  Rabbit ( GFAP secondary antibody) | Abcam | ab6799 |

**Supplementary table 2:**

**Dosimetry**

| **Doses of Radiation** | **Tissue /**  **Organs** | **M1** | **M2** | **Mean ± SEM** | **Total Absorption %** |
| --- | --- | --- | --- | --- | --- |
| **10 cGy** | **Muscle** | 8.96 | 9.87 | 9.4 ± 0.46 | **94** |
|  | **Liver** | 8.98 | * | 9.0 | **90** |
|  | **Lung** | 8.42 | 7.9 | 8.2 ± 0.26 | **82** |
|  | **Heart** | 6.30 | 7.36 | 6.8 ± 0.53 | **68** |
|  | **Brain** | 0.92 | 0.76 | 0.8 ± 0.08 | **8** |
| **50 cGy** | **Muscle** | 42.7 | 45.6 | 44 ± 1.46 | **88** |
|  | **Liver** | 43.9 | 39 | 41 ± 2.44 | **82** |
|  | **Lung** | 30.6 | 11.6 | 21 ± 9.51 | **42** |
|  | **Heart** | 24.0 | 19 | 22 ± 2.50 | **44** |
|  | **Brain** | 4.3 | 4 | 4 ± 0.15 | **8** |
| **100 cGy** | **Muscle** | 85.9 | 83.8 | 85 ± 1.04 | **85** |
|  | **Liver** | 87.6 | 87.1 | 87 ± 0.26 | **87** |
|  | **Lung** | 67.3 | 68.7 | 68 ± 0.69 | **68** |
|  | **Heart** | 64.5 | 62.1 | 63 ± 1.19 | **63** |
|  | **Brain** | 3.9 | 4.0 | 4 ± 0.05 | **4** |
| **250 cGy** | **Muscle** | 275.3 | 274.2 | 275 ± 0.57 | **100** |
|  | **Liver** | 258 | 257 | 258 ± 0.50 | **100** |
|  | **Lung** | 174.0 | 160.0 | 167 ± 7 | **66.8** |
|  | **Heart** | 190 | 165 | 178 ± 12.5 | **71.2** |
|  | **Brain** | 16 | 14 | 15 ± 1 | **6** |
| **500 cGy** | **Muscle** | 480.9 | * | 481 | **96.2** |
|  | **Liver** | 504.1 | 504.3 | 504 ± 0.08 | **100** |
|  | **Lung** | 351.8 | 267.7 | 310 ± 42.01 | **62** |
|  | **Heart** | 188.0 | * | 188 | **37.6** |
|  | **Brain** | 32.3 | 29 | 31 ± 1.65 | **6.2** |
| **1000 cGy** | **Muscle** | 1147.3 | * | 1147 | **100** |
|  | **Liver** | 1189.6 | 1193.4 | 1191 ± 1.89 | **100** |
|  | **Lung** | 1041.0 | 1037.7 | 1039 ± 1.67 | **100** |
|  | **Heart** | 601.1 | 747.0 | 674 ± 72.9 | **67.4** |
|  | **Brain** | 68.3 | 71.8 | 70 ± 1.80 | **7** |

The thermoluminescent output is expressed as output per unit weight (nC/mg).
